# Reprogramming the Canine Glioma Microenvironment with Tumor Vaccination plus Oral Losartan and Propranolol Induces Objective Responses

**DOI:** 10.1158/2767-9764.CRC-22-0388

**Published:** 2022-12-19

**Authors:** Dylan T. Ammons, Amanda Guth, Aaron J. Rozental, Jade Kurihara, Angela J. Marolf, Lyndah Chow, John F. Griffin, Rebecca Makii, Brittany MacQuiddy, Mary-Keara Boss, Daniel P. Regan, Chad Frank, Stephanie McGrath, Rebecca A. Packer, Steven Dow

**Affiliations:** 1 Department of Microbiology, Immunology and Pathology, Colorado State University, Fort Collins, Colorado.; 2 Department of Clinical Sciences, Colorado State University, Fort Collins, Colorado.; 3 Department of Environmental and Radiological Health Sciences, Colorado State University, Fort Collins, Colorado.; 4 Department of Large Animal Clinical Sciences, Texas A&M University, College Station, Texas.

## Abstract

**Purpose:**

Malignant gliomas have a highly immune-suppressive tumor microenvironment (TME) which renders them largely unresponsive to conventional therapeutics. Therefore, the current study evaluated a therapeutic protocol designed to overcome the immune barrier by combining myeloid cell–targeted immunotherapy with tumor vaccination.

**Experimental Design:**

We utilized a spontaneously occurring canine glioma model to investigate an oral TME modifying immunotherapy in conjunction with cancer stem cell (CSC) vaccination. Dogs were treated daily with losartan (monocyte migration inhibitor) and propranolol (myeloid-derived suppressor cell depleting agent) plus anti-CSC vaccination on a biweekly then monthly schedule. Tumor volume was monitored by MRI and correlated with patient immune responses.

**Results:**

Ten dogs with histologically confirmed gliomas were enrolled into a prospective, open-label clinical trial to evaluate the immunotherapy protocol. Partial tumor regression was observed in 2 dogs, while 6 dogs experienced stable disease, for an overall clinical benefit rate of 80%. Overall survival times (median = 351 days) and progression-free intervals (median = 163 days) were comparable with prior studies evaluating surgical debulking followed by immunotherapy. Dogs with detectable anti-CSC antibody responses had an increased overall survival time relative to dogs that did not generate antibody responses (vaccine responder MST = 500 days; vaccine nonresponder MST = 218 days; *P* = 0.02).

**Conclusions:**

These findings suggest that combining myeloid cell–targeted oral immunotherapy with tumor vaccination can generate objective tumor responses, even in the absence of conventional therapy. Overall, this approach has promise as a readily implemented therapeutic strategy for use in patients with brain cancer.

**Significance:**

In a pilot study of 10 dogs with glioma, we found that orally administered losartan and propranolol plus vaccination induced durable tumor responses in 8 of 10 treated dogs. The immunotherapy protocol was well tolerated, without systemic or local toxicities. These findings indicate that continuous oral immunotherapy plus tumor vaccination is a promising new strategy for glioma management that can be readily applied in clinical trials.

## Introduction

Malignant gliomas are an aggressive tumor of the brain that carry a dismal prognosis for long-term survival. For instance, humans diagnosed with a grade III glioma exhibit a median progression-free interval (mPFI) of 11 weeks and a median overall survival time (MST) of 39 weeks (1). Of note, there are remarkable similarities in survival time, clinical symptoms, and histologic properties between human and canine malignant gliomas (2, 3). The similarities in disease progression and the accessibility of canine patients makes the dog spontaneous glioma model valuable for investigation of novel therapeutics, with the potential for accelerated translation to human medicine (4, 5).

In recent years, immunotherapy has come to the forefront of anticancer therapeutics, with the blockade of immune-suppressive checkpoint pathways being adopted as a leading treatment for a variety of cancers. Such checkpoint blocking immunotherapies are highly effective in a subset of cancers but have proven to be largely ineffective for treatment of adult and pediatric gliomas (6). The lackluster responses in brain tumors are partly due to the immunologically cold nature of the malignancy, which is a result of a highly immune-suppressive tumor microenvironment (TME) that inhibits antitumor responses (7). Therefore, recent efforts for brain cancer treatment have focused on strategies to modify the TME, with the goal of enhancing the efficacy of existing immunotherapies.

In the current study, we targeted myeloid cells for depletion from the TME using two repurposed drugs: losartan and propranolol. The choice of these two drugs was based on our previous demonstration that losartan can block inflammatory monocyte migration and the findings of other groups which indicate that propranolol can inhibit myeloid-derived suppressor cell (MDSC) mobilization and immune suppression (8–10). When losartan is administered in a continuous manner, its off-target activity as a C-C chemokine receptor type 2 antagonist blocks inflammatory monocyte migration and can eventually lead to depletion of tumor-associated macrophages (8). The continuous administration of propranolol has been demonstrated to interact with the β-2 adrenergic receptor on myeloid cells to inhibit STAT3 signaling and suppress the mobilization and activation of MDSCs (9, 10). Importantly, it has been reported that both drugs reach effective concentrations in cerebrospinal fluid and brain tissues (11, 12). Therefore, to disrupt the immune-suppressive TME, we used daily losartan and propranolol to deplete tumor-resident myeloid cell populations in dogs with glioma.

In addition to TME modification, we used an allogeneic vaccination strategy to generate tumor-specific immune responses. The allogeneic vaccine consisted of pooled lysates derived from three non-glioma canine cancer cell lines. The cell lines were screened for cancer stem cell (CSC) antigen expression and selected on the basis of their enriched CSC antigen levels. This vaccine strategy was chosen because gliomas are reported to have a high abundance of CSCs and to be dependent on this population for growth and spread (13, 14). To upregulate CSC antigen expression, the cell lines used in this study were cultured in nonadherent conditions with cytokine supplementation and serum-free growth medium (15). Finally, to maximize vaccine immunogenicity, we delivered the lysate using cationic liposomes complexed with TLR-3/9 agonists (polyinosinic-polycytidylic acid and CpG oligonucleotides), which we have previously shown effectively cross-primes CD8 T-cell responses to protein antigens (16, 17).

In the current study, 10 dogs with histologically confirmed gliomas were enrolled into a trial evaluating the impact of combined TME disruption and therapeutic cancer vaccination. All animals in the study were continuously treated with losartan and propranolol, while also receiving biweekly and then monthly immunizations with a canine CSC vaccine. Throughout the study, serial MRI and clinical assessments were completed to monitor the impact of combined immunotherapy on tumor growth and animal survival. Key study findings were that the immunotherapy protocol led to an 80% clinical benefit rate, with 2 animals achieving partial tumor regression and 6 dogs experiencing stable disease. Importantly, overall survival times of study dogs were comparable with those reported in patients with canine glioma that underwent surgical tumor debulking prior to vaccination (18). Thus, our findings provide guidance for the use of repurposed TME modifying drugs combined with other immunotherapies for the treatment of gliomas.

## Materials and Methods

### Study Design and Enrollment Criteria

This study was designed as a prospective, open-label clinical trial to enroll dogs with newly diagnosed gliomas. All studies were approved by the Colorado State University (CSU) Institutional Animal Care and Use Committee and the CSU Clinical Review Board. All dog owners provided informed consent prior to enrollment into the trial.

Inclusion criteria included a diagnostic MRI indicating the presence of a supratentorial lesion consistent with a glioma. For inclusion in this article, histologic confirmation of glioma was required with 4 study dogs being histologically diagnosed postmortem (trial 1A, dogs 1–4), and 6 study dogs being histologically diagnosed before treatment using biopsy tissues (trial 1B, dogs 5–10) acquired with a neuronavigation system (Brainsight; Rogue Research; NICO Myriad, NICO Corporation). Study dogs were screened and confirmed to be free of concurrent medical conditions, including hypertension and renal dysfunction.

Exclusion criteria included the presence of other cancers or prior treatment of the glioma. To avoid immunosuppressive effects, any dog that was actively receiving corticosteroids was not enrolled until their corticosteroid dosage was reduced to less than 0.5 mg/kg/day for at least 72 hours prior to vaccination.

### Losartan and Propranolol Dosing Schedule

**FIGURE 1:**
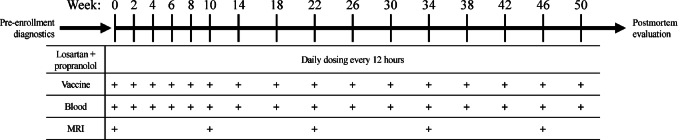
Study design. Dogs diagnosed with a histologically confirmed glioma were selected for inclusion into the trial. A total of 10 dogs were enrolled and received daily losartan plus daily propranolol with biweekly vaccination for the first 10 weeks then monthly through the duration of survival. After the first 10 weeks, blood collection and MRI was completed every three months throughout the duration of the study. Following death, a necropsy was completed.

All study dogs were treated with orally administered high-dose losartan (10 mg/kg) and propranolol (dogs 1–6 received 0.5 mg/kg; dogs 7–10 received 1 mg/kg); both administered every 12 hours (Fig. 1). Blood pressure was monitored throughout the study to ensure that hypotension did not develop. Complete blood count and serum biochemical panels were performed at study initiation, 1 month, and 3 months to assess renal and hepatic function.

### Tumor Cell Lines and Screening for High CSC Marker Expression

A series of 13 canine tumor cell lines were screened for upregulation of CSC markers following culture in tumor spheroid medium. The cell lines were obtained from the Flint Animal Cancer Center tumor cell line bank or were established in our laboratory (15). All tumor cell lines were confirmed to be of canine origin and *Mycoplasma* free. Tumor cell lines were initially maintained in Gibco Minimum Essential Medium (MEM) containing 10% FBS, 1% l-glutamine, and 1% penicillin/streptomycin (Thermo Fisher Scientific; Peak Serum Inc.). After 7 days of expansion, cells were transferred to ultra-low attachment plates (Corning) and cultured in spheroid medium. Spheroid generating medium consisted of DMEM/Ham's F-12 Nutrient Mixture (Thermo Fisher Scientific), B-27 Supplement (Thermo Fisher Scientific), 20 ng/mL basic FGF (Peprotech), 20 ng/mL EGF (Peprotech), 2 μg/mL heparin (Sigma-Aldrich), and penicillin/streptomycin (Thermo Fisher Scientific). Medium changes were completed every other day for a total of 7 days in spheroid medium.

To quantify CSC antigen expression, each cell line was screened by flow cytometry for expression of eight CSC markers (CD90, CD44, CD133, CD34, CD24, Sca1, CD117, and Oct3/4), as described previously (19). From this screen, three cell lines [Jenny (melanoma), Gracie (osteosarcoma), and Bliley (transitional cell carcinoma)] with highest CSC marker expression were selected for use in the allogeneic CSC vaccine (Supplementary Fig. S1).

### Preparation of CSC Cell Lysate Vaccines

Following a 7-day period of growth in spheroid medium, the spheroids were collected by gravity settling, washed with PBS, then lysed using distilled water. Tumor cell lysates were generated through five freeze-thaw cycles in liquid nitrogen, and then three sonication cycles on ice. Lysates were passed through 0.2 μm sterile filters (Pall) and resuspended in distilled water. The protein concentration was quantified using a bicinchoninic acid kit (Thermo Fisher Scientific) and the vaccine was prepared by pooling equivalent amounts of spheroid lysate (166.6 μg) generated from each of the three cell lines. The pooled tumor lysate was combined with a cationic liposomal-TLR3/9 vaccine adjuvant, as described previously, to generate the vaccine (17, 20). Briefly, each vaccine consisted of 500 μg pooled tumor lysate complexed to 500 μL liposome-TLR3/9 adjuvant, in a total volume of 2 mL diluent. Vaccines were then lyophilized and stored at −80°C until administered.

### Immunization Schedule

Vaccines were reconstituted with 2 mL of sterile water then delivered via two 1 mL subcutaneous injections split between each flank. After the initial immunization, vaccines were administered on weeks 2, 4, 6, 8, and 10 then once monthly until death or withdrawal from study (Fig. 1). Vaccine reactions were monitored by evaluation of the injection site and an owner questionnaire.

### Tumor Monitoring by MRI and Clinical Evaluation

Serial MRI evaluations were conducted prior to treatment (trial 1A) or after tumor biopsy (trial 1B) to obtain baseline tumor volume measurements and subsequently at 3-month intervals throughout the duration of the study period. MRI data were acquired on a 1.5 T GE Scanner (Signa HDxt) running 24.x software, using a standard human knee coil. Acquisition protocols followed the consensus recommendations on standardized MRI protocols for multicenter canine brain tumor clinical trials (4). This included transverse, sagittal, and dorsal plane T1-weighted pre- and post-contrast sequences; transverse and sagittal T2-weighted sequences; transverse GRE, FLAIR, and DTI sequences; and Three-dimensional (3D) isotropic T1-weighted images. All T1-weighted sequences were evaluated pre- and post-contrast administration. 3D T1 anatomic images were performed using the FSPGR BRAVO sequence (FOV = 25.6 cm, matrix = 256 × 256, flip angle = 12°, TR = 8.19 ms, TE = 3.39 ms, TI = 450 ms, slice thickness = 1.0 mm, NEX = 1).

Image measurements were made by veterinary radiologists, using either Philips IntelliSpace (v.4.4.551, Philips Medical) or Osirix MD (v.10.0.4, Pixmeo SARL). The evaluating radiologists were blinded to study date and dog identity when analyzing the imaging data and the software platform used for analysis was consistent over time for an individual dog. Linear measurements were made in all three dimensions on the post-contrast T1-weighted images with width measured on the transverse plane, height measured on the sagittal plane, and length measured on the dorsal plane including both contrast enhancing and non-contrast enhancing lesion components. The largest diameter and its orthogonal dimension were recorded. Volumetric measurements were made in transverse plane post-contrast T1-weighted images. When applicable, the volume calculation was corrected for interslice gap. Progression of disease was determined on the basis of tumor volume calculations according to the response assessment in neuro-oncology (RANO)/immunotherapy response assessment in neuro-oncology (iRANO) criteria, using a 40% volume increase as disease progression, a 65% reduction of tumor volume as a partial response, and all responses in between were classified as stable disease (21, 22). Progression-free interval (PFI) was calculated using the date in which the MRI measured tumor volume was greater than 40% larger than baseline tumor volume. Dogs that achieved stable disease for 3 months on protocol were counted toward the clinical benefit rate. For cases in which the dog did not survive to the first follow-up MRI (*n* = 1) the PFI was recorded as the date of death.

### Measurement of Vaccine-induced Antibody Responses to Canine Glioma Cells

A canine glioma cell line (J3T, a generous gift of Dr. Peter Dickinson, University of California Davis College of Veterinary Medicine, Davis, CA) was propagated under standard plastic adherence conditions (parental) using complete MEM (10% FBS, 1% l-glutamine, and 1% penicillin/streptomycin) or under spheroid-forming conditions, as described above. Parental cells were detached by trypsinization, while spheroids were disaggregated using Accumax (Innovative Cell Technologies, Inc.) to generate single-cell suspensions.

For quantification of antiglioma antibody titers, serum from study dogs obtained prior to vaccination and 4 weeks after the first vaccination was evaluated using a flow cytometric assay. Serial dilutions were made at 4-fold steps then serum solutions were incubated for 60 minutes at room temperature in FACS buffer (5% FBS plus 0.1% sodium azide in PBS). A rabbit anti-dog IgG H&L-FITC antibody (Jackson Laboratories, catalog no. 304-095-003, RRID:AB_2339350) was added at a dilution of 1:200 and incubated for 30 minutes at room temperature. Finally, 5 μL of 7-AAD viability dye (Thermo Fisher Scientific) was added, then cells were run on a Beckman Coulter Gallios Flow Cytometer. All serum samples were analyzed by two independent endpoint titers. Endpoint cutoff was determined to be a value less than the average plus 3.5 times the standard deviation (SD) of at least 6 blanks.

### Western Blotting to Assess Antigen Recognition by Serum from Vaccinated Dogs

Protein lysate used in the CSC vaccine (15 μg per lane) was electrophoresed under reducing conditions into a 4%–15% Mini-PROTEAN TGX gel (Bio-Rad) then transferred to a polyvinylidene difluoride membrane and blocked with 5% bovine serum albumin (BSA) in Tris-Buffered Saline, 0.1% Tween 20 (TBST) (Bio-Rad). Serum obtained prior to and 1-month after the first immunization was used at a dilution of 1:100,000 in 5% BSA to probe the membrane. An horseradish peroxidase (HRP)-conjugated rabbit anti-dog IgG H&L (Jackson Laboratories, catalog no. 304-035-003, RRID: AB_2339344) antibody at 1:100,000 was used to detect bound canine antibodies. Finally, the membrane was imaged using Clarity ECL substrate on a ChemiDoc gel imaging system (Bio-Rad). All paired samples were analyzed under the same exposure conditions.

### Assessment of Antibody Recognition of Canine Glioma Cells by Confocal Microscopy

J3T spheroid cells were fixed for 30 minutes with 4% paraformaldehyde (Thermo Fisher Scientific) then embedded in optimal cutting temperature embedding medium as described previously (23). Sectioned spheroids were blocked with 5% normal rabbit serum (Jackson Laboratories) in immunofluorescence buffer (0.2% Triton X-100, 0.1% BSA, 0.05% Tween 20 in PBS). Trial dog serum obtained 4 weeks after enrollment was then diluted 1:500 in immunofluorescence buffer and incubated overnight at 4°C. The following day, a rabbit anti-dog IgG H&L-FITC antibody (Jackson Laboratories) was diluted 1:200 and added for 1 hour at room temperature. Background fluorescence was quenched with a 30-minute incubation in 10 mmol/L copper sulfate + 50 mmol/L ammonium acetate solution then counter stained with DAPI (1 μg/mL; Sigma-Aldrich). After immunolabeling, slides were visualized using an Olympus IX3 confocal microscope and processed using Olympus cellSens software.

### Evaluation of Immune Infiltrates in Tumor Biopsies from Study Dogs and Untreated Dogs with Glioma

Formalin-fixed, paraffin-embedded (FFPE) tumor tissues were obtained from 4 study dogs (all study dogs that had tissue samples available) and from a reference group of 14 dogs with untreated gliomas. IHC was performed on FFPE tissues using a Leica BOND-MAX Fully Automated IHC Staining System, with canine cross-reactive antibodies: mouse monoclonal anti-human CD3 (Leica Biosystems, catalog no. PA0554, RRID:AB_10554454, clone LN10) and mouse monoclonal anti-human Myeloid/Histiocyte antigen (Bio-Rad, catalog no. MCA874GA, RRID:AB_324314, clone MAC387). Antigen retrieval was performed using Leica Epitope Retrieval Solution 2 (Tris-EDTA buffer, pH 9) for 20 minutes at 95°C. Detection was performed with PowerVision IHC Detection Systems (Leica Biosystems, Inc.), using a polymeric HRP anti-mouse IgG and DAB chromogen (CD3) or a polymeric alkaline phosphatase anti-mouse IgG and Fast Red chromogen (MAC387).

Whole slide brightfield images of IHC-stained slides were digitally captured using an Olympus VS120 slide scanner at 20× magnification and fixed exposure times for all samples. Quantitative image analysis was performed using Visiopharm software and visually confirmed by a veterinary pathologist (D.P. Regan). The density of tumor-infiltrating immune cells was calculated as number of immune cells per mm^2^ of viable tumor tissue.

### Gene Expression Analysis by NanoString

Tumor biopsies were evaluated histologically to confirm that at least 50% of the sample contained tumor cells. Four 10-μm sections from each sample were used for RNA extraction with an RNeasy FFPE Mini kit (QIAGEN) then evaluated with RNA High Sensitivity assays on the Qubit 2.0 Fluorometer (Invitrogen/LifeTechnologies) and 5200 Fragment Analyzer Automated CE System (Agilent). NanoString (NanoString Technologies) gene expression analysis was performed using the NanoString Canine immune-oncology (IO) panel (24). NanoString data were collected using the nCounter Analysis System at the University of Arizona Genetics Core, software 4.0.1.8. Gene expression count data were preprocessed using nSolver software (NanoString) then normalized to housekeeping genes and batch effect regressed using panel standards. Differential gene expression analysis was completed using DESeq2 with corrections for multiple comparisons (25). For corrected *P* values, a threshold of 0.1 was used to determine statistical significance.

### Statistical Analysis

Comparisons of overall survival times and PFIs between dog signalment and tumor parameters were completed using multiple univariate tests, with statistical significance determined using a likelihood-ratio test. For comparisons between two groups, Wilcoxon two-sample *t* tests were utilized. When making comparisons between prevaccine and postvaccine clinical samples, we used paired analysis, while all other comparisons used nonpaired tests. Unless specified otherwise, statistical testing that resulted in a *P* value of 0.05 or lower was considered statistically significant. All analysis was completed using R software and data were visualized using the R packages ggplot2 and survminer (26–28).

### Data Availability

The data presented in this study are available from the corresponding author, S. Dow, upon reasonable request. The R code used to analyze data in the current study is available on GitHub https://github.com/dyammons/CanineGliomaManuscript (DOI: 10.5281/zenodo.7381224).

## Results

### Study Dog Demographics

Ten dogs, consisting of 8 oligodendrogliomas and 2 astrocytomas, were enrolled into the clinical trial. Tumors were present in various brain regions with both high (*n* = 6) and low (*n* = 4) grade gliomas represented (Table 1; Supplementary Table S1). The average age at enrollment was 7.5 years old, and there were 6 male and 4 female dogs. The breed distribution of the study dogs was skewed toward brachycephalic breeds, which is consistent with the literature, as this population is known to have a predilection to develop gliomas (29, 30).

**TABLE 1:** Study demographics and objective response to therapeutic intervention

Dog ID	Age (years)	Sex	Breed	Tumor location	Glioma subclassification	Grade	Trial^b^	Time to maximal response (months)	Maximal response (%)	Objective response
1	7	FS	Boxer	Parietal	Oligodendroglioma	High	1A	3	−58	Stable
2	8	MI	Boxer	Temporal	Astrocytoma	Low	1A	3	−18	Stable
3	8	FS	Boxer	Temporal	Oligodendroglioma	Low	1A	3	−44	Stable
4^a^	8	MC	English Bulldog	Parietal	Oligodendroglioma	High	1A	0	Progression	Progression
5	5	MC	French bulldog	Frontal, Parietal	Oligodendroglioma	High	1B	3	−28	Stable
6	6	FS	Mixed	Parietal, Occipital	Oligodendroglioma	High	1B	3	+43	Progression
7	9	MC	Boston Terrier	Temporal	Oligodendroglioma	High	1B	6	−92	Partial
8	8	MI	Swiss Mountain	Parietal	Astrocytoma	Low	1B	3	−60	Stable
9	8	FS	Boxer	Right Pyriform	Oligodendroglioma	High	1B	6	−88	Partial
10	8	MC	Boxer	Right Pyriform	Oligodendroglioma	Low	1B	3	−9	Stable

^a^Denotes the dog succumbed to disease before follow-up MRI.

^b^Trial 1A dogs had a postmortem histological diagnosis, while 1B received a biopsy pre-enrollment to confirm the diagnosis.

Two study dogs were withdrawn due to progressive clinical signs but were followed to determine date of death (dog 7 withdrawn on day 272; dog 8 withdrawn on day 264). Both of the dogs that were withdrawn early, transitioned to a therapeutic protocol which included radiotherapy with continued daily losartan and propranolol plus monthly vaccination. Survival data from these two dogs were censored at the date of first radiotherapy. On follow-up, dog 8 survived for 13 months and dog 7 survived for 15 months. All other dogs remained on protocol until succumbing to disease.

### Clinical Responses to Treatment

**FIGURE 2:**
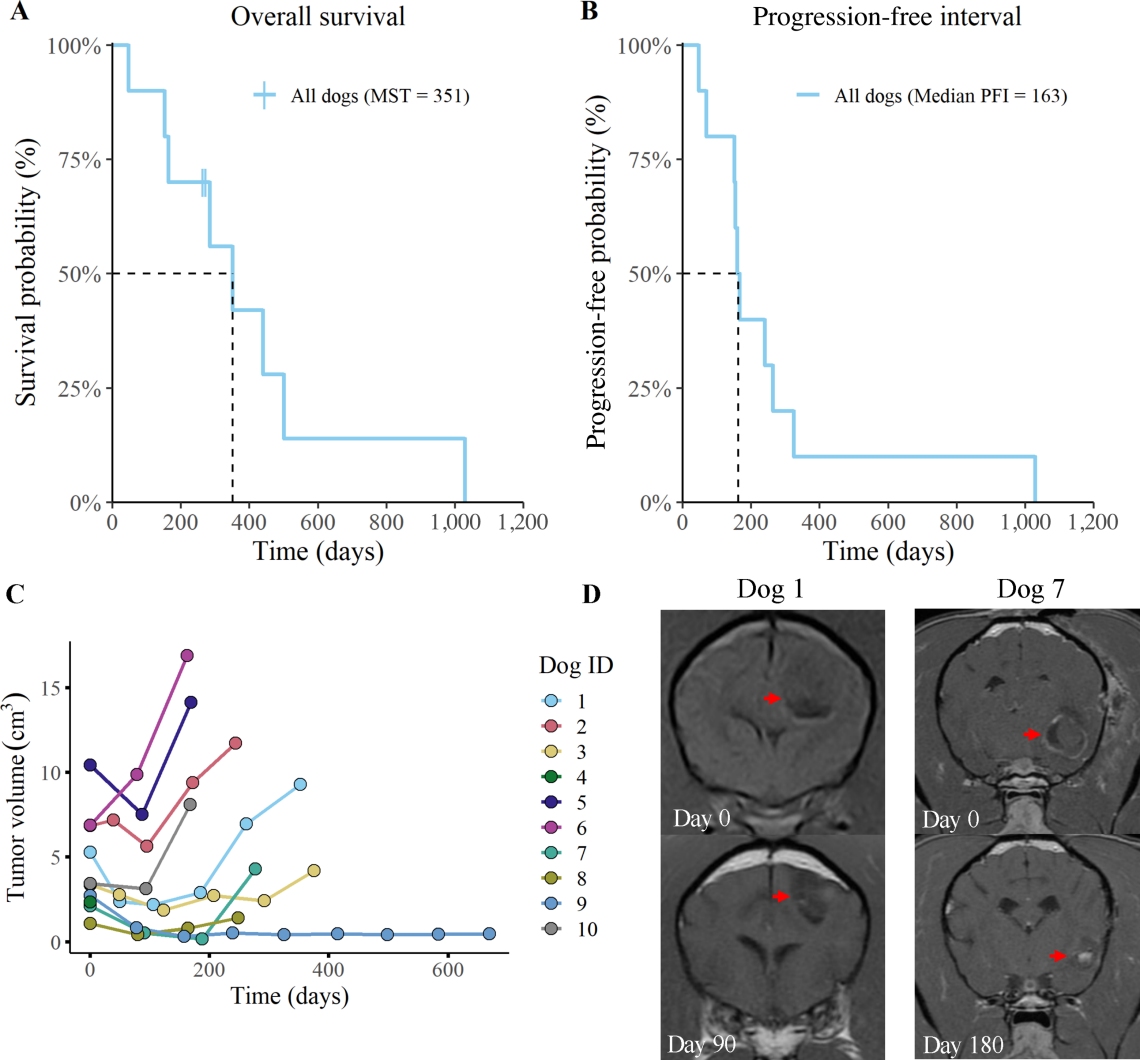
Overall survival and PFI curves for all enrolled dogs. Overall survival (**A**) and PFI (**B**) curves for all 10 dogs are depicted. Two dogs were censored from the overall survival data following deviation from the treatment protocol (time of censorship is noted with a vertical hash mark). Plot (**C**) represents tumor volume measurements, as determined by MRI. Tumor volume was evaluated every 3 months and volume was determined using transverse plane post-contrast T1-weighted images. Measurements were recorded until death or withdrawal from study. Images (**D**) are representative of changes in tumor volume for one dog with stable disease (dog 1) and one dog with partial tumor regression (dog 7).

Using the MRI-based RANO/iRANO classification scheme of objective tumor responses, we determined that 2 dogs exhibited rapid disease progression, 6 had stable disease, and 2 demonstrated a partial tumor response (Table 1). All dogs reached their maximal response within 6 months of starting the therapeutic intervention. Overall survival time was calculated on the basis of the time that the dogs survived following administration of the first vaccine. The median survival time for study dogs was 351 days, with a 1-year survival rate of 42% (Fig. 2A). In addition to overall survival, the PFI was determined to be 163 days from time of initial vaccination to MRI tumor volume progression or death (Fig. 2B). Tumor growth curves revealed that 4 dogs (dogs 3, 7, 8, and 9) exhibited a tumor volume reduction (44%, 92%, 60%, and 88%; respectively) following the first immunization and maintained their positive response. Although dogs 7 and 8 had a sustained tumor volume reduction, they showed signs of neurologic progression and were withdrawn to receive supplemental radiotherapy. Three other dogs (dogs 1, 2, and 10) exhibited a rapid relapse after an initial tumor volume reduction (Fig. 2C and D). In addition, 1 dog (dog 4) rapidly succumbed to progressive disease. Finally, we observed that dogs with an initial tumor volume of less than 5 cm^3^ had a tendency to exhibit sustained tumor regression following immunotherapy. This observation suggests that dogs with smaller tumor burdens may be better candidates for the immunotherapy only protocol. Overall, these data indicate that combination immunotherapy led to stabilization of tumor growth in a majority of dogs, while a smaller subset achieved partial tumor regression.

A secondary objective of this study was to evaluate the safety of the immunotherapy protocol. Notably, there were no detectable adverse events associated with daily oral administration of losartan or propranolol throughout the duration of the study, and we did not observe hypotension in any of the study dogs. We observed grade 1 adverse events in 5 dogs following at least one immunization which were limited to minor skin irritation at the injection site that resolved without treatment. All other dogs exhibited no detectable adverse reactions to vaccination.

### Evaluation of CSC Tumor Vaccine Immunogenicity

**FIGURE 3:**
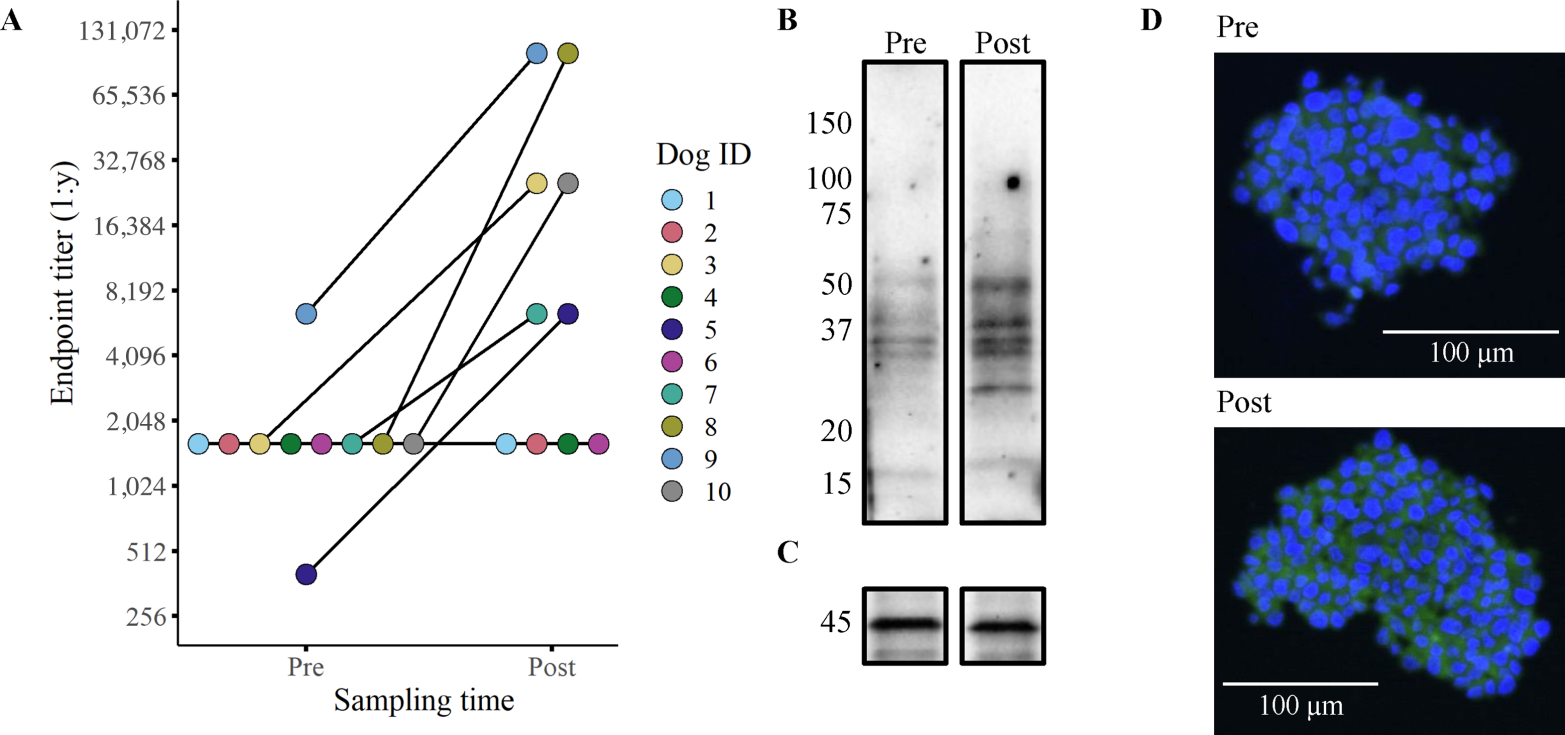
Six of 10 dogs exhibited detectable antiglioma humoral responses. Humoral responses to vaccination were quantified using flow cytometry–based endpoint titers with disaggregated J3T spheroids (**A**). Representative Western blots (**B/C**) and confocal micrographs (**D**) depicting the serum antibody binding in a dog before (pre) immunization and 1-month post-vaccination (post). The Western blots show binding to proteins found in the vaccine lysate (serum from dog 7) with the lower blot (**C**) depicting β-actin loading control. The confocal images tested binding of antibodies to J3T spheroids (**D**; serum from dog 3). All serum samples used in the assays were obtained after two vaccinations (week 4).

We next evaluated antibody titers following CSC vaccination, as a means of assessing overall immunotherapy responses. The primary evaluation of vaccine response was completed using flow cytometry–based quantitative endpoint titers to detect the binding of circulating immunoglobulins to J3T (a canine glioma cell line) spheroids (31). Evaluation of J3T spheroid endpoint titers revealed that 4 dogs were nonresponders (undetectable anti-J3T antibodies) and that 6 dogs exhibited detectable increases in anti-J3T antibodies postvaccination (Fig. 3A). We also determined that J3T cells cultured under spheroid conditions were more immunogenic than the same cells cultured as plastic adherent cells, which indicates specificity to antigens only present on spheroid cells (Supplementary Fig. S2).

Pre- and post-immunization serum samples were also screened by Western blotting to evaluate reactivity to proteins in the vaccine. The banding patterns from 5 humoral responders revealed consistent protein recognition patterns between dogs (Fig. 3B and C; Supplementary Fig. S3). The most highly conserved protein bands appeared at molecular weights of 50 and 37 kDa. To further investigate antibody responses, we complete immunocytochemistry of J3T spheroids which revealed recognition of both intracellular and surface proteins (Fig. 3D; Supplementary Fig. S4). These findings indicate that the tumor lysate vaccine induced broad recognition of multiple glioma antigens.

Finally, the duration of antibody responses was evaluated in a subset of 5 study dogs using serum samples obtained throughout the study. Notably, the antibody titers remained consistent with minimal fluctuation in antibody binding over the months evaluated (Supplementary Fig. S5). Together, these data indicate that vaccination produced durable antiglioma humoral immune responses, and that evaluation of antibody titers could be used to predict clinical responses to the combined immunotherapy protocol.

### Correlation of Immune Response to Immunotherapy with Clinical Outcomes

**FIGURE 4:**
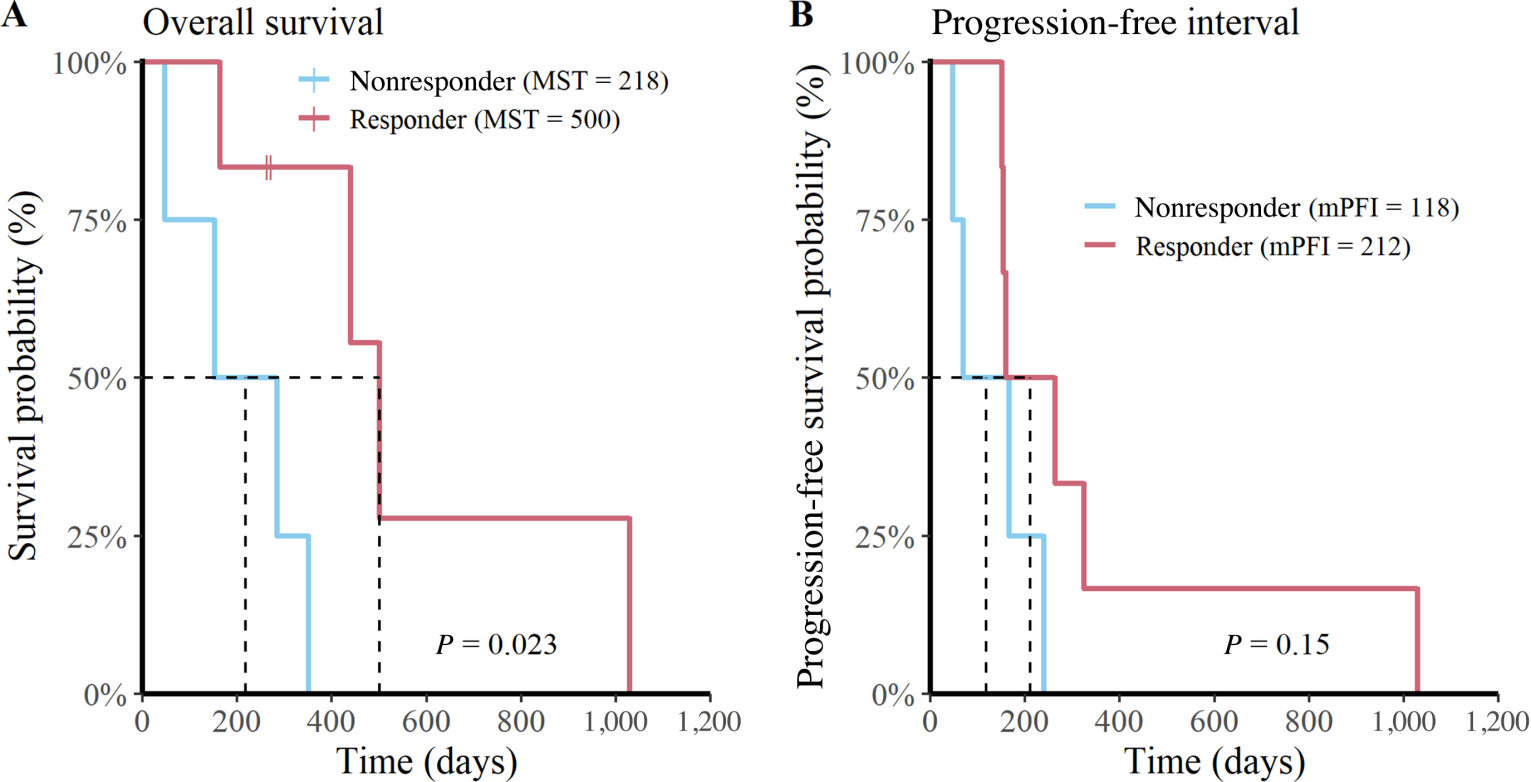
Dogs with detectable humoral responses have an increased overall survival rate. Depiction of overall survival (**A**) and PFI (**B**) curves stratified for vaccine responders (*n* = 6; red) and nonresponders (*n* = 4; blue) for all 10 dogs.

To determine whether antibody titers correlated with tumor responses, we stratified overall survival and PFI data by vaccine antibody status (i.e., vaccine responder vs. nonresponder) then completed univariate analysis. The analysis revealed that dogs with a detectable antiglioma antibody response had enhanced overall survival times relative to nonresponders (*P* = 0.02; responder MST = 500 days; nonresponder MST = 218 days; Fig. 4A). When PFI was subjected to the same analysis, we found that the correlation with vaccine status did not reach a level of statistical significance, though numerically the mPFI was longer in the responder cohort (*P* = 0.15; responder mPFI = 212 days; nonresponder mPFI = 118 days; Fig. 4B).

To further evaluate clinical correlates with humoral responses, we compared the maximal tumor response (percent change in tumor volume) between dogs classified as vaccine responders and nonresponders (Supplementary Fig. S6). This analysis failed to reach statistical significance (*P* = 0.11) but provided further data to correlate clinical outcomes with humoral status. Finally, we evaluated other factors that could have influenced study dog outcomes. This analysis was completed using multiple univariate analysis on key strata (breed, sex, tumor grade, and trial) and none of the tests revealed evidence of statistically significant correlations with clinical outcome measures (Supplementary Table S2). Therefore, vaccine antibody response was the only factor identified in this study that correlated significantly with clinical outcomes. Overall, these findings suggest that vaccine response was the key variable associated with increased survival in dogs treated with this novel immunotherapy combination.

### Assessment of Tumor Immune Infiltrates in Study Dogs Compared with Untreated Canine Gliomas

Finally, we used postmortem tumor biopsy tissues to compare the densities of immune infiltrates and the transcriptomes of trial dogs relative to an untreated cohort of dogs with gliomas. In total, we evaluated 4 dogs from the trial (dogs 1, 2, 3, and 6) and 14 dogs from an untreated reference group. The reference group consisted of 10 oligodendrogliomas, 2 astrocytomas, and 2 undefined glioma tumor types. Seven of the tumors were classified as high grade and seven were classified as low grade. The 4 study dogs used for analysis consisted of one dog who exhibited a vaccine response and had partial tumor regression while the other 3 dogs did not appear to respond to intervention. Therefore, the tissues collected from the treated dogs might not fully recapitulate the immunologic changes observed in dogs who responded to intervention.

**FIGURE 5:**
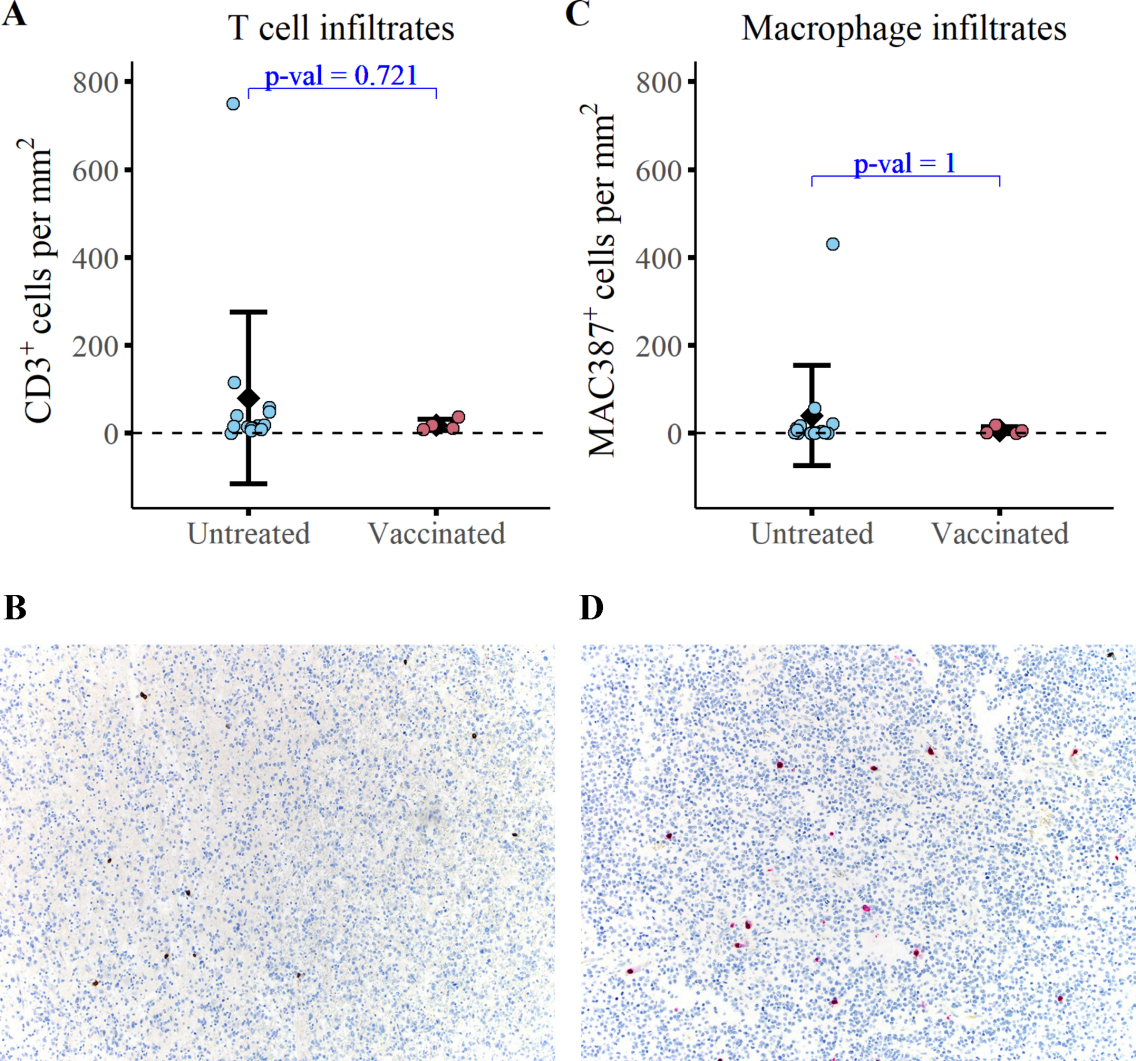
Immunized dogs exhibit no detectable change in tumor-infiltrating immune cells relative to untreated controls at the time of necropsy. Quantification of T-cell infiltrates (CD3^+^; **A**) and macrophage infiltrates (MAC387^+^; **C**) at the time of necropsy in 4 study dogs and 14 untreated dogs with gliomas. Representative images of CD3 immunolabeling (**B**) and MAC387 immunolabeling (**D**) are depicted. A Wilcoxon two-sample *t* test was conducted to evaluate statistical significance.

IHC analysis revealed that the mean density of tumor-infiltrating CD3^+^ T cells was 19.0 ± 12.6 cells/mm^2^ in study dogs compared with 79.9 ± 195 cells/mm^2^ in reference dogs (Fig. 5A and B; ±SD). The average density of macrophage tumor infiltrates (MAC387^+^) was 6.4 ± 8.2 cells/mm^2^ in study dogs compared with 39.9 ± 114 cells/mm^2^ in the reference dogs (Fig. 5C and D; ±SD). Statistically, the mean tumor-infiltrating T-cell and macrophage densities were not significantly different between study dogs and control dogs. Notably, the sparse densities of immune infiltrates are consistent with the densities typical of human glioma (32, 33). Overall, these findings support the classification of canine gliomas as immunologically “cold” tumors.

We next completed immune transcriptome analysis to investigate whether there were changes in immune gene expression profiles between treated and untreated gliomas. These studies employed the canine IO NanoString panel and used DESeq2 to complete differential gene expression analysis (24). Analysis indicated there were no statistically significant differences in the transcriptome between treated and untreated tumors. To further analyze the dataset, we completed covariate analysis to investigate how sex, tumor type, and tumor grade impacted gene expression. This analysis revealed statistically significant changes in gene expression between dogs with high-grade and low-grade tumors (28 genes upregulated, 3 genes downregulated; Supplementary Fig. S7). Of note, gene set enrichment analysis revealed that genes associated with PD-1 and CD28 signaling were increased in high-grade tumors relative to low-grade gliomas. The enrichment of these pathways provides evidence that there may be more exhausted T cells present in high-grade gliomas compared with low-grade tumors. To investigate further, we compared the abundances of T cell and macrophage tumor infiltrates and found no detectable differences between high- and low-grade gliomas (Supplementary Fig. S8). These findings indicate that the transcriptomic changes are likely due to functional differences in T cells rather than changes in T-cell abundance. Overall, these data provide a better understanding of the immune landscape of canine gliomas and suggest that canine gliomas recapitulate human gliomas in terms of their overall immune responses.

## Discussion

In the current study, we assessed a novel combination immunotherapy for generation of antitumor activity in dogs with spontaneously arising gliomas. Importantly, the canine glioma model is considered to be an excellent large animal model for human glioma and is valuable in the investigation of translational therapeutics (34, 35). Key study findings include generation of partial tumor regression in 20% of dogs, along with durable tumor responses (mPFI, 163 days) in most animals. The median overall survival time (351 days) reported in this study is comparable with survival times previously reported in dogs treated with debulking surgery plus vaccination (MST = 212 days), or with debulking surgery plus chemotherapy (MST = 240 days; refs. 18, 36). In the current study, tumor responses correlated with vaccine antibody responses, as dogs that mounted detectable vaccine responses experienced significantly longer overall survival times than dogs with undetectable antibody responses (responder MST = 500 days; nonresponder MST = 218 days). Taken together, these results indicate that the combination of TME disruption using losartan and propranolol with allogeneic tumor vaccination, can elicit substantial antitumor activity in dogs with gliomas.

The prognosis for patients diagnosed with glioma is dismal and there is a need for effective therapeutic interventions in both human and canine patients. In dogs, definitive treatment for glioma consists of surgical debulking, radiotherapy, and/or chemotherapy (37, 38). Dogs that receive some form of definitive treatment have been reported to have a median survival time of 84 days, while dogs that only receive palliative treatment have a 26-day MST (39). Recently, novel immunotherapy interventions have been explored in the canine glioma model. One such immunotherapy for canine glioma used a CD200 agonist, autologous vaccination, and conjunctive surgical debulking to achieve a median survival time of 12.7 months (∼387 days; ref. 40). The CD200 trial only evaluated high-grade glioma cases, which limits direct comparison with the current trial results. Nonetheless, the median survival time observed in the current study (351 days) obtained with immunotherapy alone compares favorably with the results of combination immunotherapy plus surgery.

Limitations of the current study include a small sample size, a mix of low- and high-grade tumors, and a lack of a control arm consisting of dogs with untreated gliomas. However, prior literature indicates that the survival times for untreated dogs with glioma are short, thus all animals were treated in the current study (40, 41). Furthermore, due to the limited availability of frozen blood leukocyte samples, our study did not evaluate T-cell responses to vaccination. Nonetheless, prior studies have found that antitumor antibody responses following tumor vaccination typically correlate with T-cell responses, and therefore antibody titers can be used as a proxy for overall T-cell immune responses (42, 43). Finally, the immune density and transcriptome studies only included tumor samples from 4 study dogs, and only one was obtained from a vaccine responder, thereby skewing the analysis toward nonresponders (*n* = 3) versus untreated dogs (*n* = 14).

Analysis of tumor-infiltrating immune cell densities and transcriptomes failed to detect any statistically significant differences between glioma samples from treated (*n* = 4) and naïve (*n* = 14) dogs. As noted above, the lack of vaccine responding animals is a major limitation to this analysis. However, the failure to observe significant T-cell infiltrates in tumor samples from study dogs could also reflect the possibility that humoral immune responses may have been more important than cellular responses in this study. For example, in the 6 vaccine responding dogs, tumor control may have been primarily mediated through antibody-dependent cellular cytotoxicity, which might not be reflected in T-cell and macrophage infiltrates (44). Alternatively, it is possible that the 6 dogs who generated antibody responses were more immune competent than the nonresponders and may have exhibited enhanced clinical outcomes regardless of intervention. Nonetheless, further investigation of the mechanism(s) of action associated with combined TME modification plus vaccination is warranted.

Further analysis of immune transcriptome responses in tumor tissues revealed important grade-related changes in gene expression. We identified upregulation of key immune regulatory pathways in high-grade tumors compared with low-grade tumors, including lymphoid cell interactions, integrin interactions, and cell surface interactions at endothelial surface. Furthermore, we also found that the densities of tumor immune cell infiltrates were not significantly different between high- and low-grade tumors. Together, these findings suggest that tumor-infiltrating immune cells in high-grade tumors are likely more activated and potentially exhausted relative to low-grade tumors.

We have previously demonstrated the antitumor activity of losartan as a TME modifying drug in dogs, and other groups have noted the efficacy of propranolol as an MDSC modifying agent in rodent studies and as an antiangiogenic agent in dogs (9, 45, 46). In addition, prior studies have found that losartan and propranolol can enhance antitumor immunity and contribute to increased survival times (47, 48). Therefore, the current pilot study was not designed to investigate the antitumor activity of each component in combined protocol. Finally, a valuable aspect of using repurposed drugs (i.e., losartan and propranolol) as cancer immunotherapy agents is that the drugs are FDA approved, have strong safety records, and can be readily obtained for use in clinical trials (49).

Our study employed a whole-cell lysate vaccine derived from non-glioma canine cancer cell lines enriched for CSC antigens. We have previously reported the efficacy of similar allogeneic cell line lysate cancer vaccines in dogs with hemangiosarcoma (50). At the time of study initiation, we did not have access to canine glioma cell lines for inclusion in the vaccine. Since then, several canine glioma cell lines, including the J3T cell line used for immune assays in the current study, have been made available (31, 51). Thus, the use of canine glioma cell lines in the preparation of future allogeneic vaccines would be predicted to generate greater antitumor activity.

In summary, a novel immunotherapy protocol consisting of daily treatment with losartan and propranolol combined with tumor vaccination was well tolerated, induced partial tumor regression in 2 of 10 dogs, led to stable disease in 6 of 10 dogs, and resulted in a median overall survival time of 351 days in dogs with glioma. The overall positive results of this pilot study, obtained in a spontaneous canine glioma model, indicate the potential for such an approach to be incorporated into the design of future glioma immunotherapy trials in human patients.

## Supplementary Material

Supplemental Figures/Tables S1 Supplemental Table 1. Breakdown of study dog demographics.
Supplemental Figure 1. Spheroid culture enriches for CD44 on three canine cancer cell lines.
Supplemental Figure 2. Patient serum has minimal reactivity to parental J3T.
Supplemental Figure 3. Additional western blots from vaccinated study dogs.
Supplemental Figure 4. Additional images of spheroid ICC antibody binding.
Supplemental Figure 5. Responders exhibit durable antibody responses.
Supplemental Figure 6. Comparison of maximal response between humoral responders and non-responders.
Supplemental Table 2. Univariate analysis of key strata indicate humoral status is the only predictor of survival.
Supplemental Figure 7. Differential gene expression analysis reveals enhanced immunoreactivity in high-grade canine gliomas relative to low-grade.
Supplemental Figure 8. Tumor grade does not impact abundance of tumor infiltrating immune cells at the time of necropsy.

## References

[1] Lamborn KR , Yung WKA, Chang SM, Wen PY, Cloughesy TF, DeAngelis LM, . Progression-free survival: an important end point in evaluating therapy for recurrent high-grade gliomas. Neuro Oncol 2008;10:162–70. 18356283 10.1215/15228517-2007-062 PMC2613818

[2] Herranz C , Fernández F, Martín-Ibáñez R, Blasco E, Crespo E, De la Fuente C, . Spontaneously arising canine glioma as a potential model for human glioma. J Comp Pathol 2016;154:169–79. 26804204 10.1016/j.jcpa.2015.12.001

[3] Hubbard ME , Arnold S, Zahid AB, McPheeters M, O'Sullivan MG, . Naturally occurring canine glioma as a model for novel therapeutics. Cancer Invest 2018;36:415–23. 30234401 10.1080/07357907.2018.1514622

[4] Packer RA , Rossmeisl JH, Kent MS, Griffin IV JF, Mazcko C, . Consensus recommendations on standardized magnetic resonance imaging protocols for multicenter canine brain tumor clinical trials. Vet Radiol Ultrasound 2018;59:261–71. 29522650 10.1111/vru.12608 PMC5942214

[5] Koehler JW , Miller AD, Miller CR, Porter B, Aldape K, Beck J, . A revised diagnostic classification of canine glioma: towards validation of the canine glioma patient as a naturally occurring preclinical model for human glioma. J Neuropathol Exp Neurol 2018;77:1039–54. 30239918 10.1093/jnen/nly085 PMC6181180

[6] Brahm CG , van Linde ME, Enting RH, Schuur M, Otten RH, Heymans MW, . The current status of immune checkpoint inhibitors in neuro-oncology: a systematic review. Cancers 2020;12:586. 32143288 10.3390/cancers12030586 PMC7139638

[7] Tong N , He Z, Ma Y, Wang Z, Huang Z, Cao H, . Tumor associated macrophages, as the dominant immune cells, are an indispensable target for immunologically cold tumor—glioma therapy? Front Cell Dev Biol 2021;9:706286. 34368156 10.3389/fcell.2021.706286 PMC8337013

[8] Regan DP , Coy JW, Chahal KK, Chow L, Kurihara JN, Guth AM, . The angiotensin receptor blocker losartan suppresses growth of pulmonary metastases via AT1R-independent inhibition of CCR2 signaling and monocyte recruitment. J Immunol 2019;202:3087–102. 30971441 10.4049/jimmunol.1800619 PMC6504574

[9] Mohammadpour H , MacDonald CR, Qiao G, Chen M, Dong B, Hylander BL, . β2 adrenergic receptor–mediated signaling regulates the immunosuppressive potential of myeloid-derived suppressor cells. J Clin Invest 2019;129:5537–52. 31566578 10.1172/JCI129502 PMC6877316

[10] MacDonald C , Ministero S, Pandey M, Robinson D, Hong EF, Hylander B, . Comparing thermal stress reduction strategies that influence MDSC accumulation in tumor bearing mice. Cell Immunol 2021;361:104285. 33484943 10.1016/j.cellimm.2021.104285 PMC7883813

[11] Walle UK , Thibodeaux H, Privitera PJ, Walle T. Stereochemistry of tissue distribution of racemic propranolol in the dog. Chirality 1989;1:192–6. 2642048 10.1002/chir.530010303

[12] Li Z , Bains JS, Ferguson AV. Functional evidence that the angiotensin antagonist losartan crosses the blood-brain barrier in the rat. Brain Res Bull 1993;30:33–9. 8420632 10.1016/0361-9230(93)90036-b

[13] Zhai Y , Li G, Li R, Chang Y, Feng Y, Wang D, . Single-cell RNA-sequencing shift in the interaction pattern between glioma stem cells and immune cells during tumorigenesis. Front Immunol 2020;11:581209. 33133100 10.3389/fimmu.2020.581209 PMC7580180

[14] Piper K , DePledge L, Karsy M, Cobbs C. Glioma stem cells as immunotherapeutic targets: advancements and challenges. Front Oncol 2021;11:615704. 33718170 10.3389/fonc.2021.615704 PMC7945033

[15] Ishiguro T , Ohata H, Sato A, Yamawaki K, Enomoto T, Okamoto K. Tumor-derived spheroids: relevance to cancer stem cells and clinical applications. Cancer Sci 2017;108:283–9. 28064442 10.1111/cas.13155 PMC5378268

[16] U'ren L , Kedl R, Dow S. Vaccination with liposome–DNA complexes elicits enhanced antitumor immunity. Cancer Gene Ther 2006;13:1033–44. 16841080 10.1038/sj.cgt.7700982

[17] Zaks K , Jordan M, Guth A, Sellins K, Kedl R, Izzo A, . Efficient immunization and cross-priming by vaccine adjuvants containing TLR3 or TLR9 agonists complexed to cationic liposomes. J Immunol 2006;176:7335–45. 16751377 10.4049/jimmunol.176.12.7335

[18] Merickel JL , Pluhar GE, Rendahl A, O'Sullivan MG. Prognostic histopathologic features of canine glial tumors. Vet Pathol 2021;58:945–51. 34219560 10.1177/03009858211025795 PMC10923237

[19] Guth AM , Deogracias M, Dow SW. Comparison of cancer stem cell antigen expression by tumor cell lines and by tumor biopsies from dogs with melanoma and osteosarcoma. Vet Immunol Immunopathol 2014;161:132–40. 25146881 10.1016/j.vetimm.2014.07.006 PMC4264625

[20] Boss MK , Watts R, Harrison LG, Hopkins S, Chow L, Trageser E, . Immunologic effects of stereotactic body radiotherapy in dogs with spontaneous tumors and the impact of intratumoral OX40/TLR agonist immunotherapy. Int J Mol Sci 2022;23:826. 35055015 10.3390/ijms23020826 PMC8775899

[21] Okada H , Weller M, Huang R, Finocchiaro G, Gilbert MR, Wick W, . Immunotherapy response assessment in neuro-oncology: a report of the RANO working group. Lancet Oncol 2015;16:e534–42. 26545842 10.1016/S1470-2045(15)00088-1 PMC4638131

[22] Ellingson BM , Wen PY, Cloughesy TF. Modified criteria for radiographic response assessment in glioblastoma clinical trials. Neurotherapeutics 2017;14:307–20. 28108885 10.1007/s13311-016-0507-6 PMC5398984

[23] Gomes IC , Acquarone M, de Moraes Maciel R, Erlich RB, Rehen SK. Analysis of pluripotent stem cells by using cryosections of embryoid bodies. J Vis Exp 2010;46:2344. 10.3791/2344 PMC3159676 21178966

[24] Lenz JA , Assenmacher CA, Costa V, Louka K, Rau S, Keuler NS, . Increased tumor-infiltrating lymphocyte density is associated with favorable outcomes in a comparative study of canine histiocytic sarcoma. Cancer Immunol Immunother 2022;71:807–18. 34415404 10.1007/s00262-021-03033-z PMC8858331

[25] Love MI , Huber W, Anders S. Moderated estimation of fold change and dispersion for RNA-seq data with DESeq2. Genome Biol 2014;15:550. 25516281 10.1186/s13059-014-0550-8 PMC4302049

[26] Core Team R. R: a Language and Environment for Statistical Computing; 2020.

[27] Wickham H . Elegant graphics for data analysis. Media 2009;35:10–1007.

[28] Kassambara A , Kosinski M, Biecek P, Fabian S. Package ‘survminer’. Draw. Surviv. Curves using ‘ggplot2’(R Packag. version 0.4.9); 2017.

[29] Miller AD , Miller CR, Rossmeisl JH. Canine primary intracranial cancer: a clinicopathologic and comparative review of glioma, meningioma, and choroid plexus tumors. Front Oncol 2019;9:1151. 31788444 10.3389/fonc.2019.01151 PMC6856054

[30] Dobson JM . Breed-predispositions to cancer in pedigree dogs. ISRN Vet Sci 2013;2013:941275. 23738139 10.1155/2013/941275 PMC3658424

[31] Rainov NG , Koch S, Sena-Esteves M, Berens ME. Characterization of a canine glioma cell line as related to established experimental brain tumor models. J Neuropathol Exp Neurol 2000;59:607–13. 10901232 10.1093/jnen/59.7.607

[32] Krane GA , O'Dea CA, Malarkey DE, Miller AD, Miller CR, Tokarz DA, . Immunohistochemical evaluation of immune cell infiltration in canine gliomas. Vet Pathol 2021;58:952–63. 34196247 10.1177/03009858211023946 PMC11404454

[33] Robinson MH , Vasquez J, Kaushal A, MacDonald TJ, Vega JEV, Schniederjan M, . Subtype and grade-dependent spatial heterogeneity of T-cell infiltration in pediatric glioma. J Immunother Cancer 2020;8:e001066. 32788236 10.1136/jitc-2020-001066 PMC7422651

[34] Dickinson PJ , LeCouteur RA, Higgins RJ, Bringas JR, Larson RF, Yamashita Y, . Canine spontaneous glioma: a translational model system for convection-enhanced delivery. Neuro Oncol 2010;12:928–40. 20488958 10.1093/neuonc/noq046 PMC2940703

[35] Hicks J , Platt S, Kent M, Haley A. Canine brain tumours: a model for the human disease? Vet Comp Oncol 2017;15:252–72. 25988678 10.1111/vco.12152

[36] Crespo HE , Mariné AF, Battle MPI, Massó JFB, Feliu-Pascual AL. Survival time after surgical debulking and temozolomide adjuvant chemotherapy in canine intracranial gliomas. Vet Sci 2022;9:427. 36006342 10.3390/vetsci9080427 PMC9414206

[37] Dickinson PJ . Advances in diagnostic and treatment modalities for intracranial tumors. J Vet Intern Med 2014;28:1165–85. 24814688 10.1111/jvim.12370 PMC4857954

[38] Debreuque M , De Fornel P, David I, Delisle F, Ducerveau MN, Devauchelle P, . Definitive-intent uniform megavoltage fractioned radiotherapy protocol for presumed canine intracranial gliomas: retrospective analysis of survival and prognostic factors in 38 cases (2013–2019). BMC Vet Res 2020;16:412. 33129320 10.1186/s12917-020-02614-x PMC7603708

[39] José-López R , Gutierrez-Quintana R, de la Fuente C, Manzanilla EG, Suñol A, Pi Castro D, . Clinical features, diagnosis, and survival analysis of dogs with glioma. J Vet Intern Med 2021;35:1902–17. 34117807 10.1111/jvim.16199 PMC8295679

[40] Olin MR , Ampudia-Mesias E, Pennell CA, Sarver A, Chen CC, Moertel CL, . Treatment combining CD200 immune checkpoint inhibitor and tumor-lysate vaccination after surgery for pet dogs with high-grade glioma. Cancers 2019;11:137. 30682795 10.3390/cancers11020137 PMC6406711

[41] Rossmeisl JH , Jones JC, Zimmerman KL, Robertson JL. Survival time following hospital discharge in dogs with palliatively treated primary brain tumors. J Am Vet Med Assoc 2013;242:193–8. 23276095 10.2460/javma.242.2.193

[42] Pluhar GE , Grogan PT, Seiler C, Goulart M, SantaCruz KS, Carlson C, . Anti-tumor immune response correlates with neurological symptoms in a dog with spontaneous astrocytoma treated by gene and vaccine therapy. Vaccine 2010;28:3371–8. 20197146 10.1016/j.vaccine.2010.02.082 PMC2854671

[43] Jäger E , Nagata Y, Gnjatic S, Wada H, Stockert E, Karbach J, . Monitoring CD8 T cell responses to NY-ESO-1: correlation of humoral and cellular immune responses. Proc Natl Acad Sci U S A 2000;97:4760–5. 10781081 10.1073/pnas.97.9.4760 PMC18306

[44] Andersen BM , Pluhar GE, Seiler CE, Goulart MR, SantaCruz KS, Schutten MM, . Vaccination for invasive canine meningioma induces in situ production of antibodies capable of antibody-dependent cell-mediated cytotoxicity. Cancer Res 2013;73:2987–97. 23471847 10.1158/0008-5472.CAN-12-3366 PMC3655124

[45] Regan DP , Chow L, Das S, Haines L, Palmer E, Kurihara JN, . Losartan blocks osteosarcoma-elicited monocyte recruitment, and combined with the kinase inhibitor toceranib, exerts significant clinical benefit in canine metastatic osteosarcoma. Clin Cancer Res 2022;28:662–76. 34580111 10.1158/1078-0432.CCR-21-2105 PMC8866227

[46] Amaya CN , Perkins M, Belmont A, Herrera C, Nasrazadani A, Vargas A, . Non-selective beta blockers inhibit angiosarcoma cell viability and increase progression free-and overall-survival in patients diagnosed with metastatic angiosarcoma. Oncoscience 2018;5:109–19. 29854879 10.18632/oncoscience.413 PMC5978448

[47] Wrobel LJ , Bod L, Lengagne R, Kato M, Prévost-Blondel A, Le Gal FA. Propranolol induces a favourable shift of anti-tumor immunity in a murine spontaneous model of melanoma. Oncotarget 2016;7:77825–37. 27788481 10.18632/oncotarget.12833 PMC5363624

[48] O'Rawe M , Wickremesekera AC, Pandey R, Young D, Sim D, FitzJohn T, . Treatment of glioblastoma with re-purposed renin-angiotensin system modulators: results of a phase I clinical trial. J Clin Neurosci 2022;95:48–54. 34929651 10.1016/j.jocn.2021.11.023

[49] Murphy JE , Wo JY, Ryan DP, Clark JW, Jiang W, Yeap BY, . Total neoadjuvant therapy with FOLFIRINOX in combination with losartan followed by chemoradiotherapy for locally advanced pancreatic cancer: a phase 2 clinical trial. JAMA Oncol 2019;5:1020–7. 31145418 10.1001/jamaoncol.2019.0892 PMC6547247

[50] U'Ren LW , Biller BJ, Elmslie RE, Thamm DH, Dow SW. Evaluation of a novel tumor vaccine in dogs with hemangiosarcoma. J Vet Intern Med 2007;21:113–20. 17338158 10.1892/0891-6640(2007)21[113:eoantv]2.0.co;2

[51] Empl MT , Macke S, Winterhalter P, Puff C, Lapp S, Stoica G, . The growth of the canine glioblastoma cell line D-GBM and the canine histiocytic sarcoma cell line DH82 is inhibited by the resveratrol oligomers hopeaphenol and r2-viniferin. Vet Comp Oncol 2014;12:149–59. 22882564 10.1111/j.1476-5829.2012.00349.x

