# Supplementary material for: Reprogramming the Canine Glioma Microenvironment with Tumor Vaccination plus Oral Losartan and Propranolol Induces Objective Responses
Source: Cancer Res Commun. 2022 Dec 19;2(12):1657–67. doi: 10.1158/2767-9764.CRC-22-0388 (PMC9835010; doi:10.1158/2767-9764.CRC-22-0388)
Supplement: Supplemental Figures/Tables S1 — Supplemental Table 1. Breakdown of study dog demographics. Supplemental Figure 1. Spheroid culture enriches for CD44 on three canine cancer cell lines. Supplemental Figure 2. Patient serum has minimal reactivity to parental J3T. Supplemental Figure 3. Additional western blots from vaccinated study dogs. Supplemental Figure 4. Additional images of spheroid ICC antibody binding. Supplemental Figure 5. Responders exhibit durable antibody responses. Supplemental Figure 6. Comparison of maximal response between humoral responders and non-responders. Supplemental Table 2. Univariate analysis of key strata indicate humoral status is the only predictor of survival. Supplemental Figure 7. Differential gene expression analysis reveals enhanced immunoreactivity in high-grade canine gliomas relative to low-grade. Supplemental Figure 8. Tumor grade does not impact abundance of tumor infiltrating immune cells at the time of necropsy. [file crc-22-0388-s01.pptx]

## Slide 1
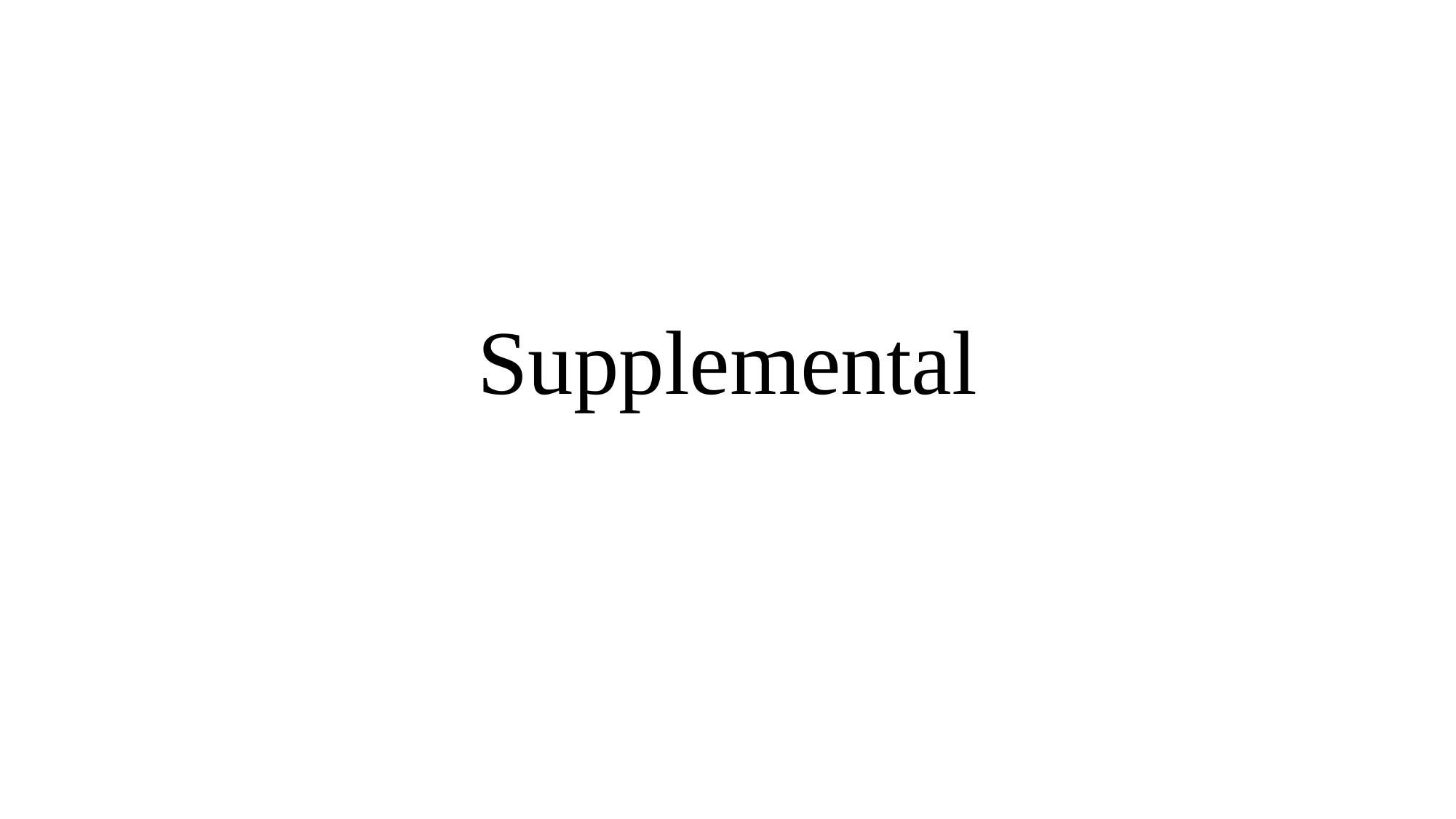

# Supplemental

## Slide 2
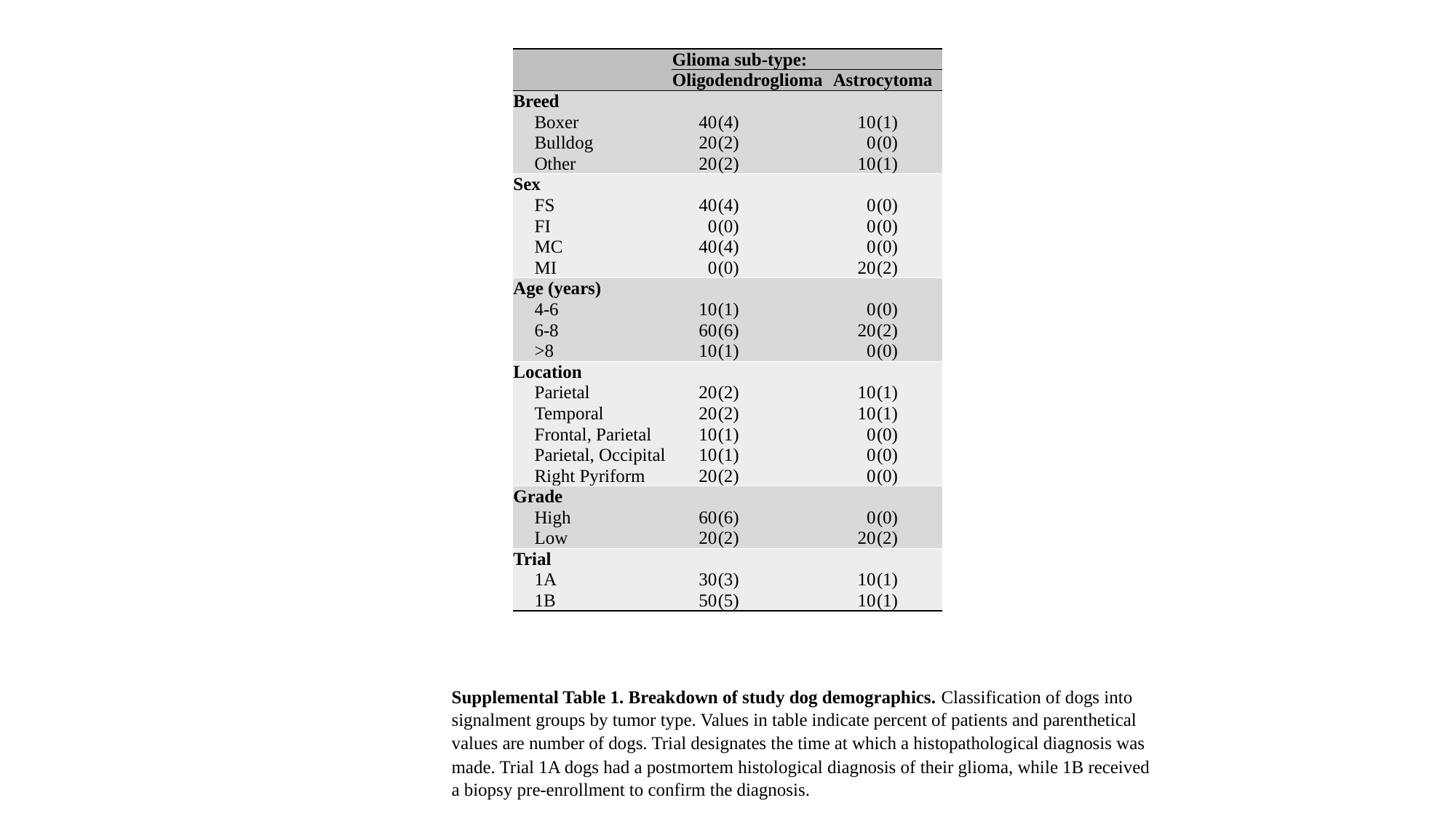

| | | Glioma sub-type: | | | |
| --- | --- | --- | --- | --- | --- |
| | | Oligodendroglioma | | Astrocytoma | |
| Breed | | | | | |
| | Boxer | 40 | (4) | 10 | (1) |
| | Bulldog | 20 | (2) | 0 | (0) |
| | Other | 20 | (2) | 10 | (1) |
| Sex | | | | | |
| | FS | 40 | (4) | 0 | (0) |
| | FI | 0 | (0) | 0 | (0) |
| | MC | 40 | (4) | 0 | (0) |
| | MI | 0 | (0) | 20 | (2) |
| Age (years) | | | | | |
| | 4-6 | 10 | (1) | 0 | (0) |
| | 6-8 | 60 | (6) | 20 | (2) |
| | >8 | 10 | (1) | 0 | (0) |
| Location | | | | | |
| | Parietal | 20 | (2) | 10 | (1) |
| | Temporal | 20 | (2) | 10 | (1) |
| | Frontal, Parietal | 10 | (1) | 0 | (0) |
| | Parietal, Occipital | 10 | (1) | 0 | (0) |
| | Right Pyriform | 20 | (2) | 0 | (0) |
| Grade | | | | | |
| | High | 60 | (6) | 0 | (0) |
| | Low | 20 | (2) | 20 | (2) |
| Trial | | | | | |
| | 1A | 30 | (3) | 10 | (1) |
| | 1B | 50 | (5) | 10 | (1) |
Supplemental Table 1. Breakdown of study dog demographics. Classification of dogs into signalment groups by tumor type. Values in table indicate percent of patients and parenthetical values are number of dogs. Trial designates the time at which a histopathological diagnosis was made. Trial 1A dogs had a postmortem histological diagnosis of their glioma, while 1B received a biopsy pre-enrollment to confirm the diagnosis.

## Slide 3
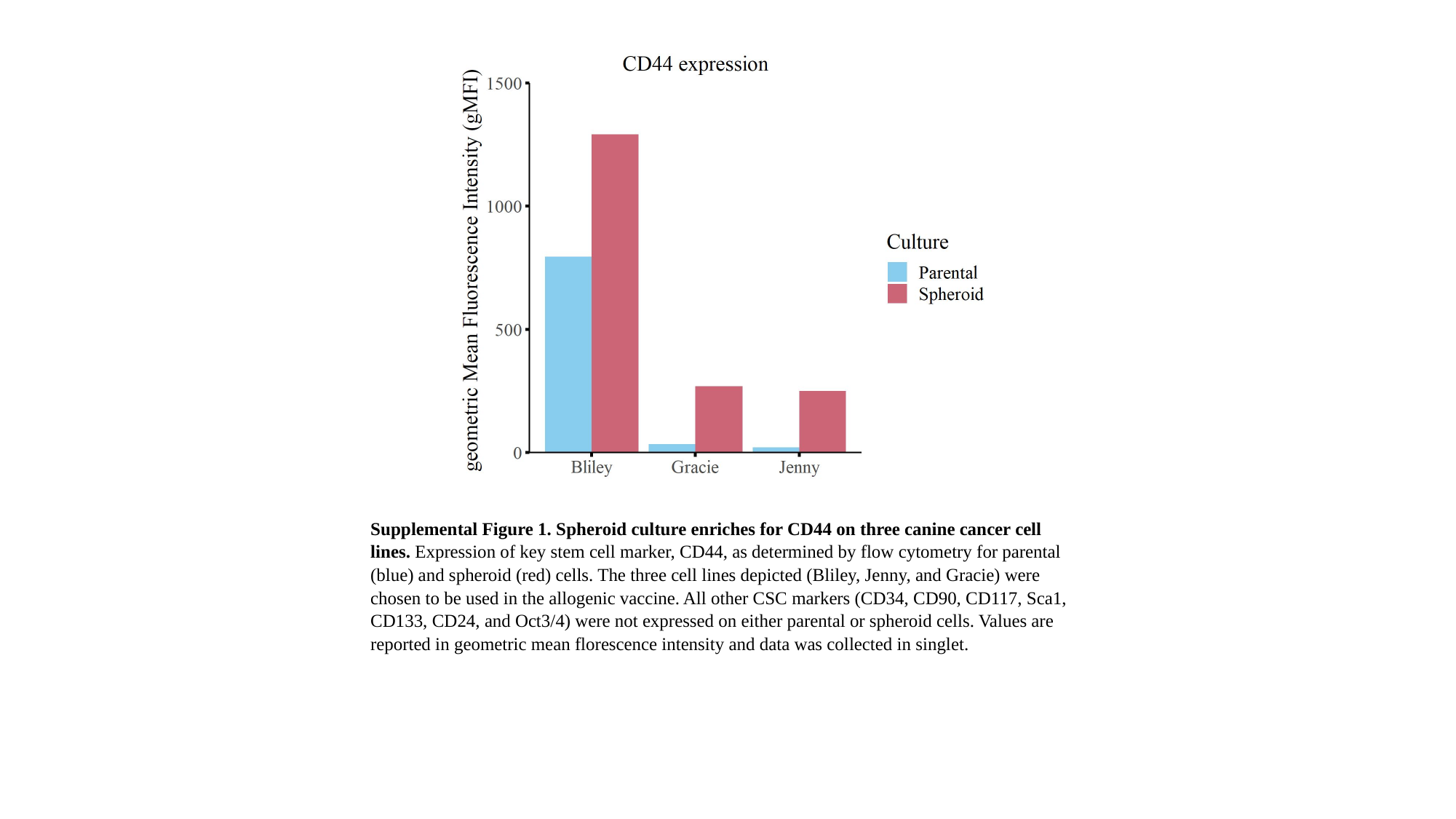

Supplemental Figure 1. Spheroid culture enriches for CD44 on three canine cancer cell lines. Expression of key stem cell marker, CD44, as determined by flow cytometry for parental (blue) and spheroid (red) cells. The three cell lines depicted (Bliley, Jenny, and Gracie) were chosen to be used in the allogenic vaccine. All other CSC markers (CD34, CD90, CD117, Sca1, CD133, CD24, and Oct3/4) were not expressed on either parental or spheroid cells. Values are reported in geometric mean florescence intensity and data was collected in singlet.

## Slide 4
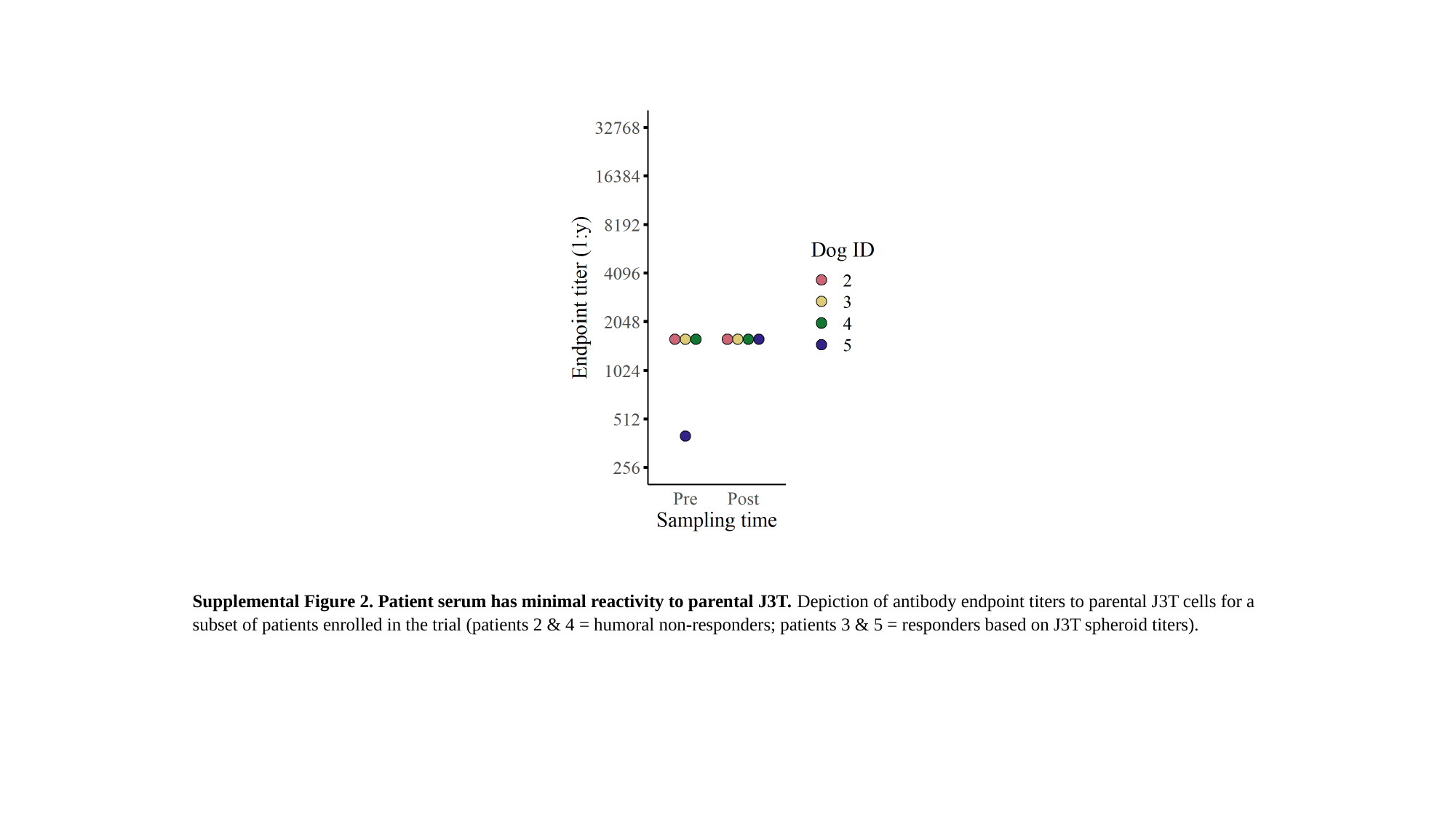

Supplemental Figure 2. Patient serum has minimal reactivity to parental J3T. Depiction of antibody endpoint titers to parental J3T cells for a subset of patients enrolled in the trial (patients 2 & 4 = humoral non-responders; patients 3 & 5 = responders based on J3T spheroid titers).

## Slide 5
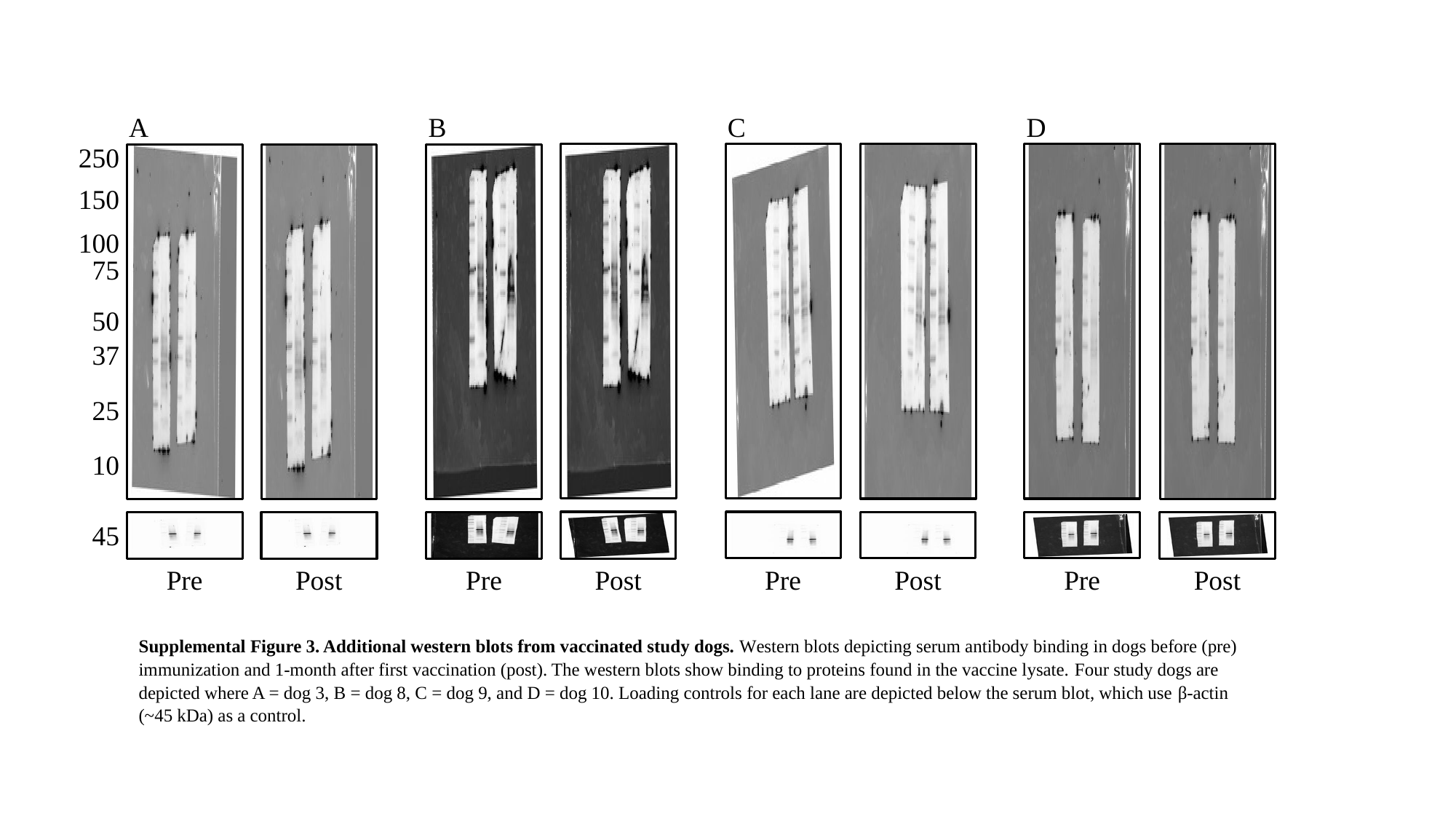

A
B
C
D
250
150
100
75
50
37
25
10
45
Pre
Post
Pre
Post
Pre
Post
Pre
Post
Supplemental Figure 3. Additional western blots from vaccinated study dogs. Western blots depicting serum antibody binding in dogs before (pre) immunization and 1-month after first vaccination (post). The western blots show binding to proteins found in the vaccine lysate. Four study dogs are depicted where A = dog 3, B = dog 8, C = dog 9, and D = dog 10. Loading controls for each lane are depicted below the serum blot, which use β-actin (~45 kDa) as a control.

## Slide 6
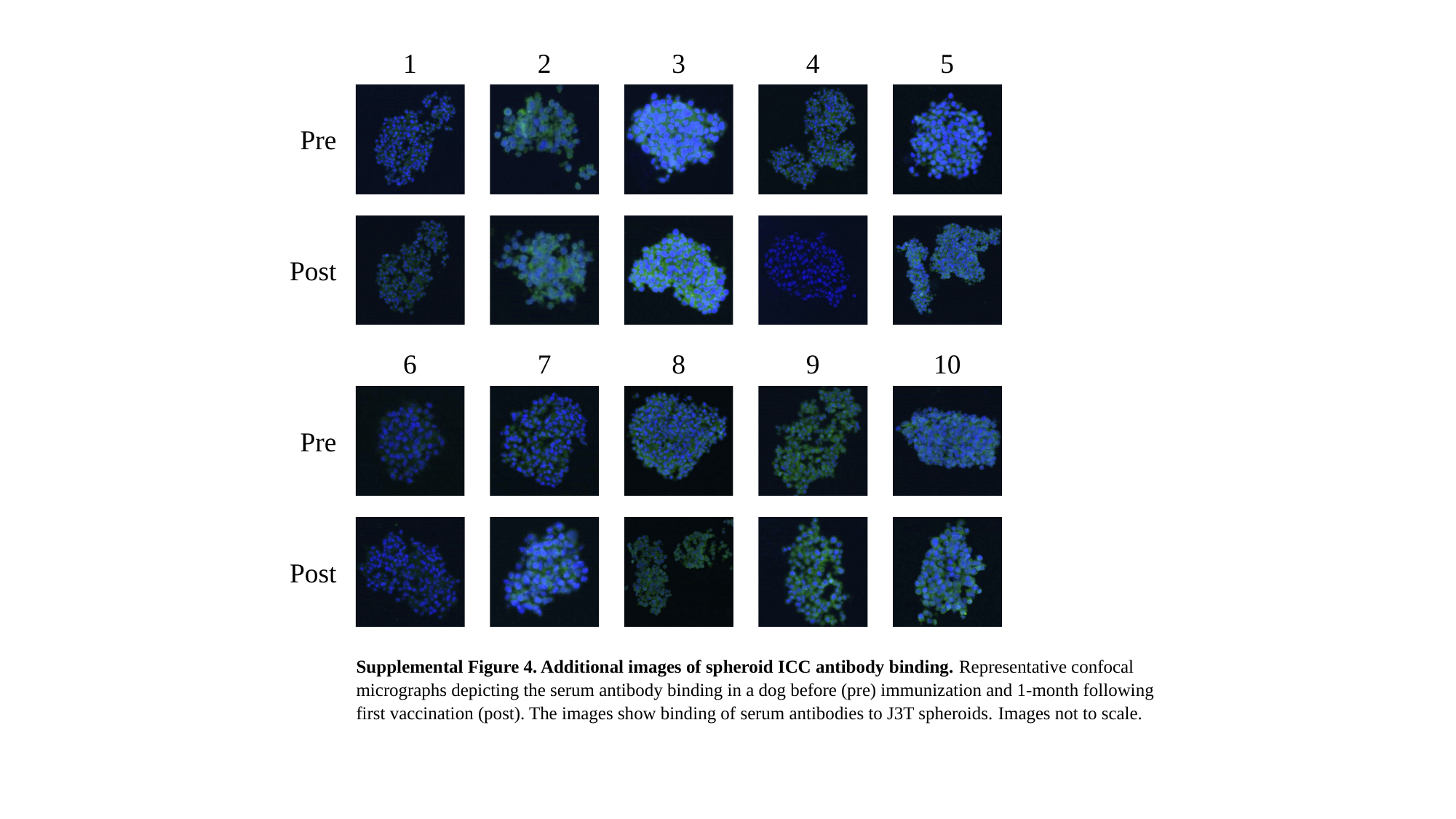

1
2
3
4
5
Pre
Post
6
7
8
9
10
Pre
Post
Supplemental Figure 4. Additional images of spheroid ICC antibody binding. Representative confocal micrographs depicting the serum antibody binding in a dog before (pre) immunization and 1-month following first vaccination (post). The images show binding of serum antibodies to J3T spheroids. Images not to scale.

## Slide 7
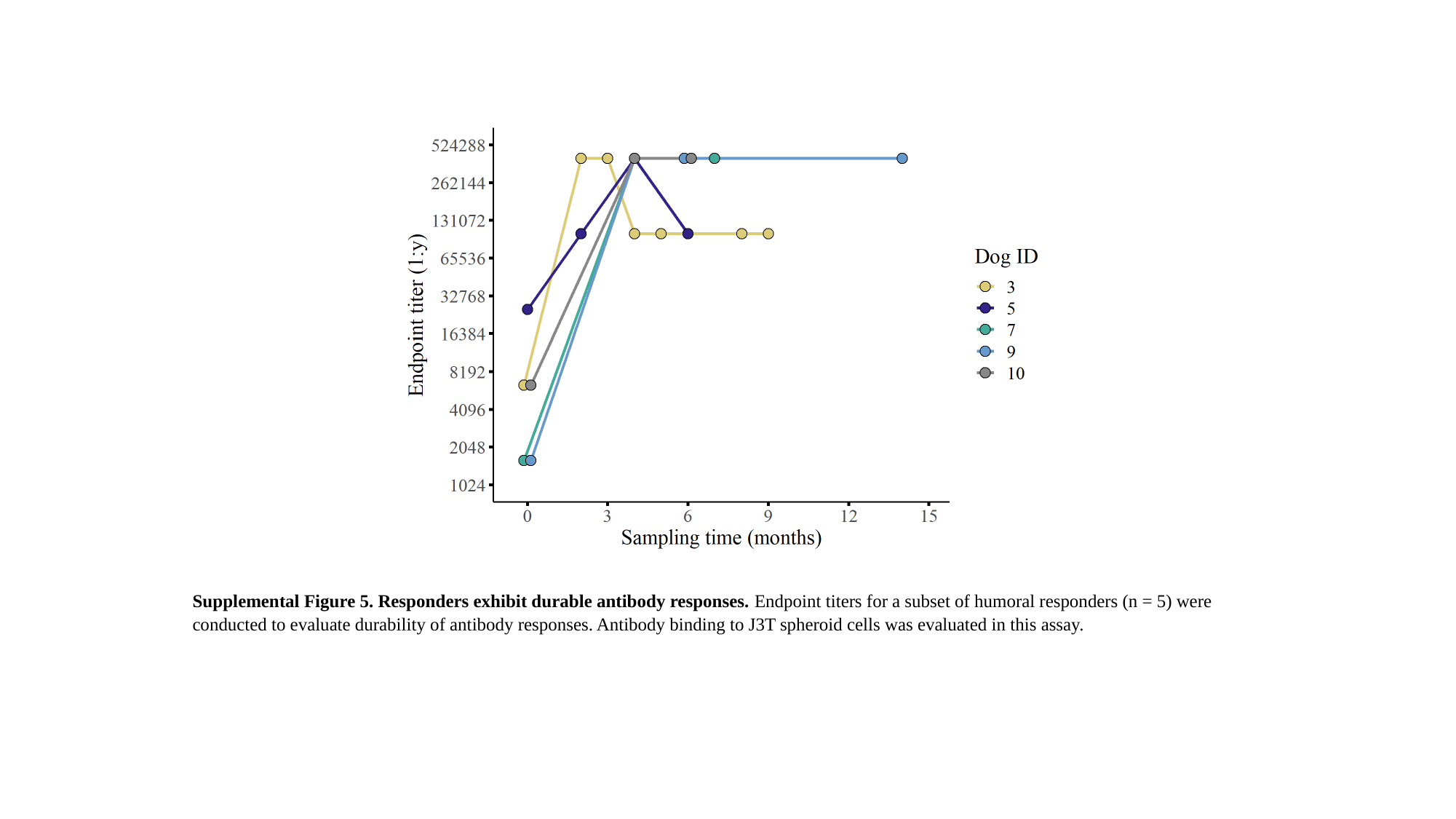

Supplemental Figure 5. Responders exhibit durable antibody responses. Endpoint titers for a subset of humoral responders (n = 5) were conducted to evaluate durability of antibody responses. Antibody binding to J3T spheroid cells was evaluated in this assay.

## Slide 8
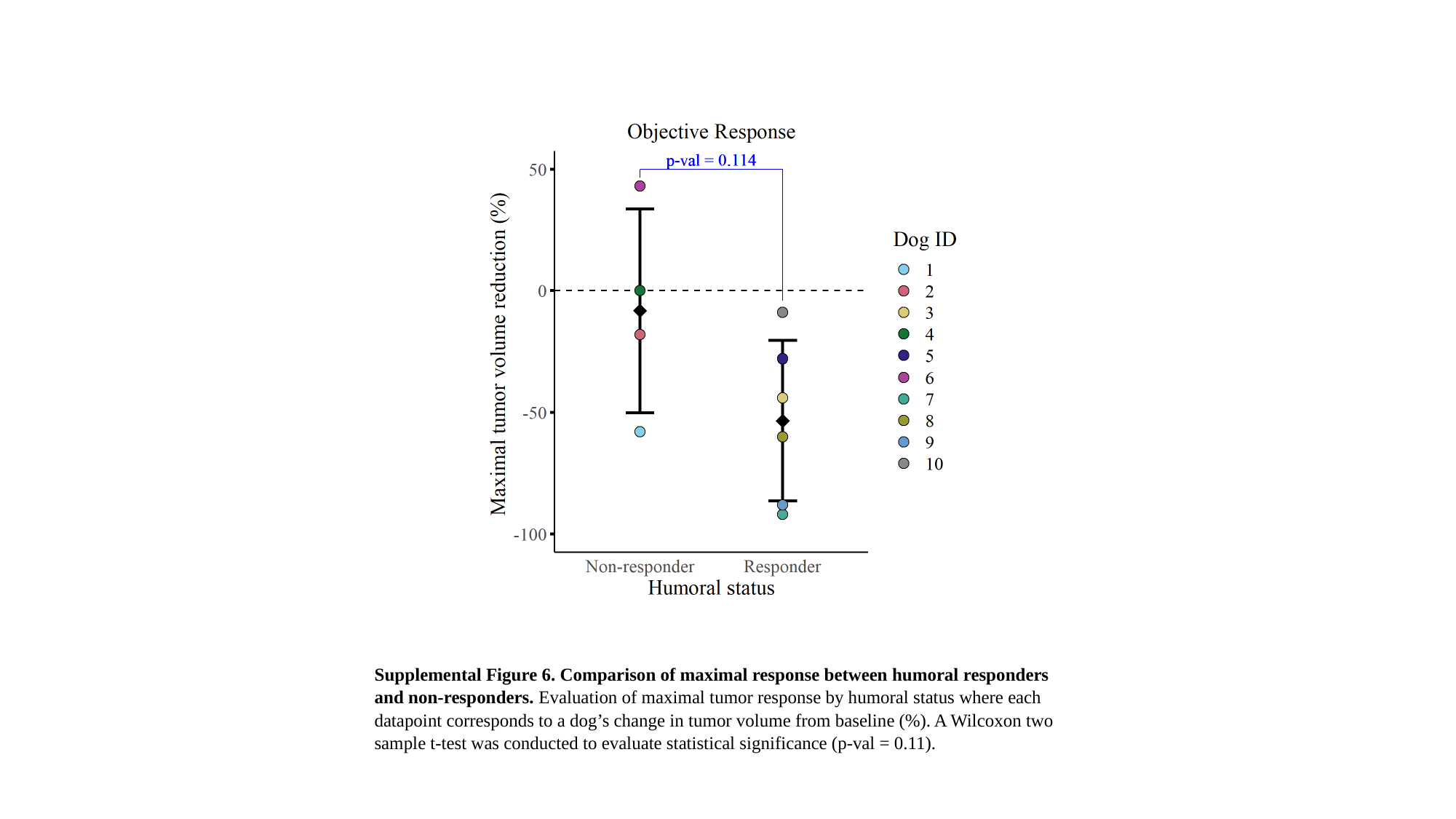

Supplemental Figure 6. Comparison of maximal response between humoral responders and non-responders. Evaluation of maximal tumor response by humoral status where each datapoint corresponds to a dog’s change in tumor volume from baseline (%). A Wilcoxon two sample t-test was conducted to evaluate statistical significance (p-val = 0.11).

## Slide 9
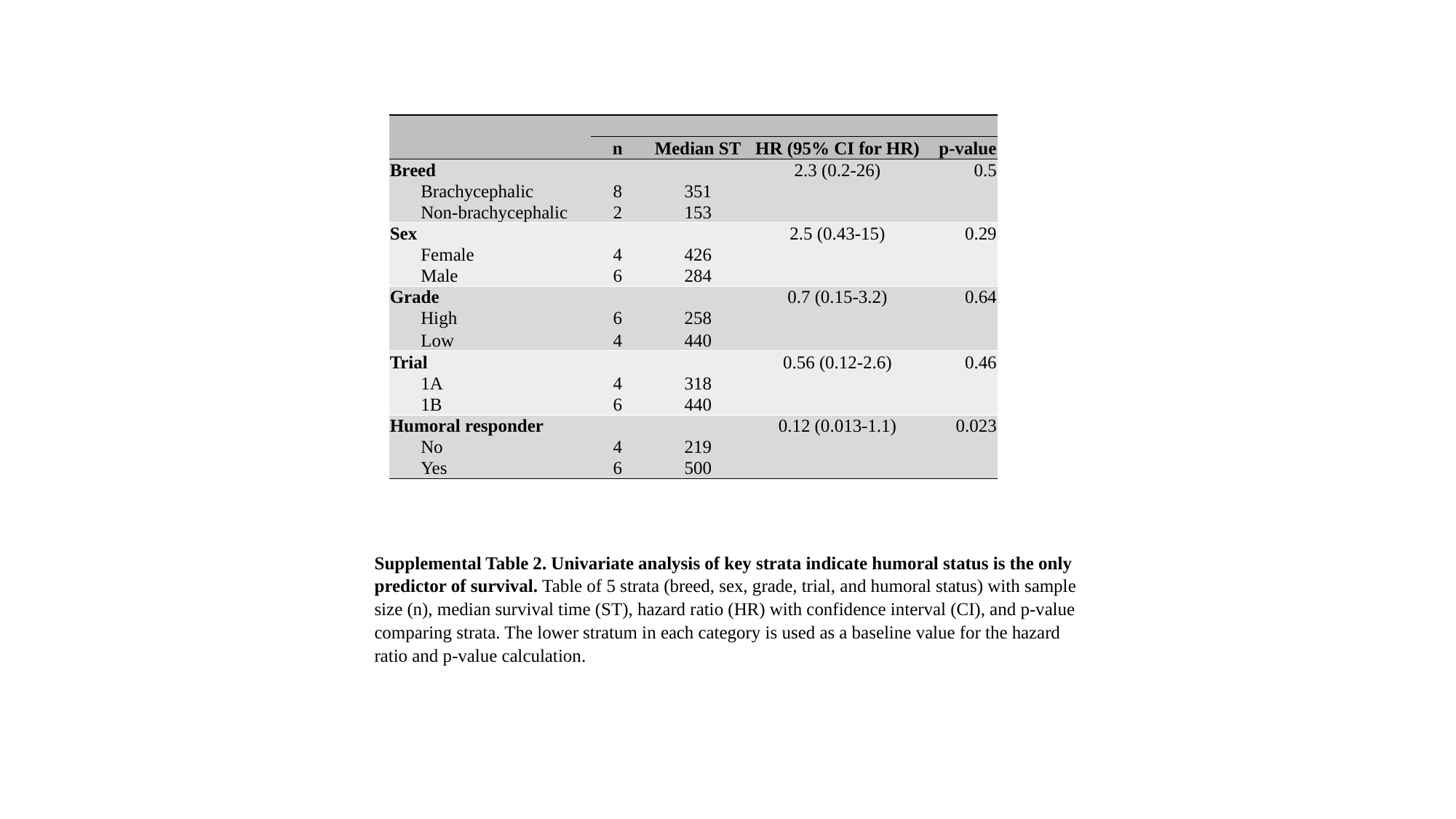

| | | | | | | |
| --- | --- | --- | --- | --- | --- | --- |
| | | | n | Median ST | HR (95% CI for HR) | p-value |
| Breed | | | | | 2.3 (0.2-26) | 0.5 |
| | Brachycephalic | | 8 | 351 | | |
| | Non-brachycephalic | | 2 | 153 | | |
| Sex | | | | | 2.5 (0.43-15) | 0.29 |
| | Female | | 4 | 426 | | |
| | Male | | 6 | 284 | | |
| Grade | | | | | 0.7 (0.15-3.2) | 0.64 |
| | High | | 6 | 258 | | |
| | Low | | 4 | 440 | | |
| Trial | | | | | 0.56 (0.12-2.6) | 0.46 |
| | 1A | | 4 | 318 | | |
| | 1B | | 6 | 440 | | |
| Humoral responder | | | | | 0.12 (0.013-1.1) | 0.023 |
| | No | | 4 | 219 | | |
| | Yes | | 6 | 500 | | |
Supplemental Table 2. Univariate analysis of key strata indicate humoral status is the only predictor of survival. Table of 5 strata (breed, sex, grade, trial, and humoral status) with sample size (n), median survival time (ST), hazard ratio (HR) with confidence interval (CI), and p-value comparing strata. The lower stratum in each category is used as a baseline value for the hazard ratio and p-value calculation.

## Slide 10
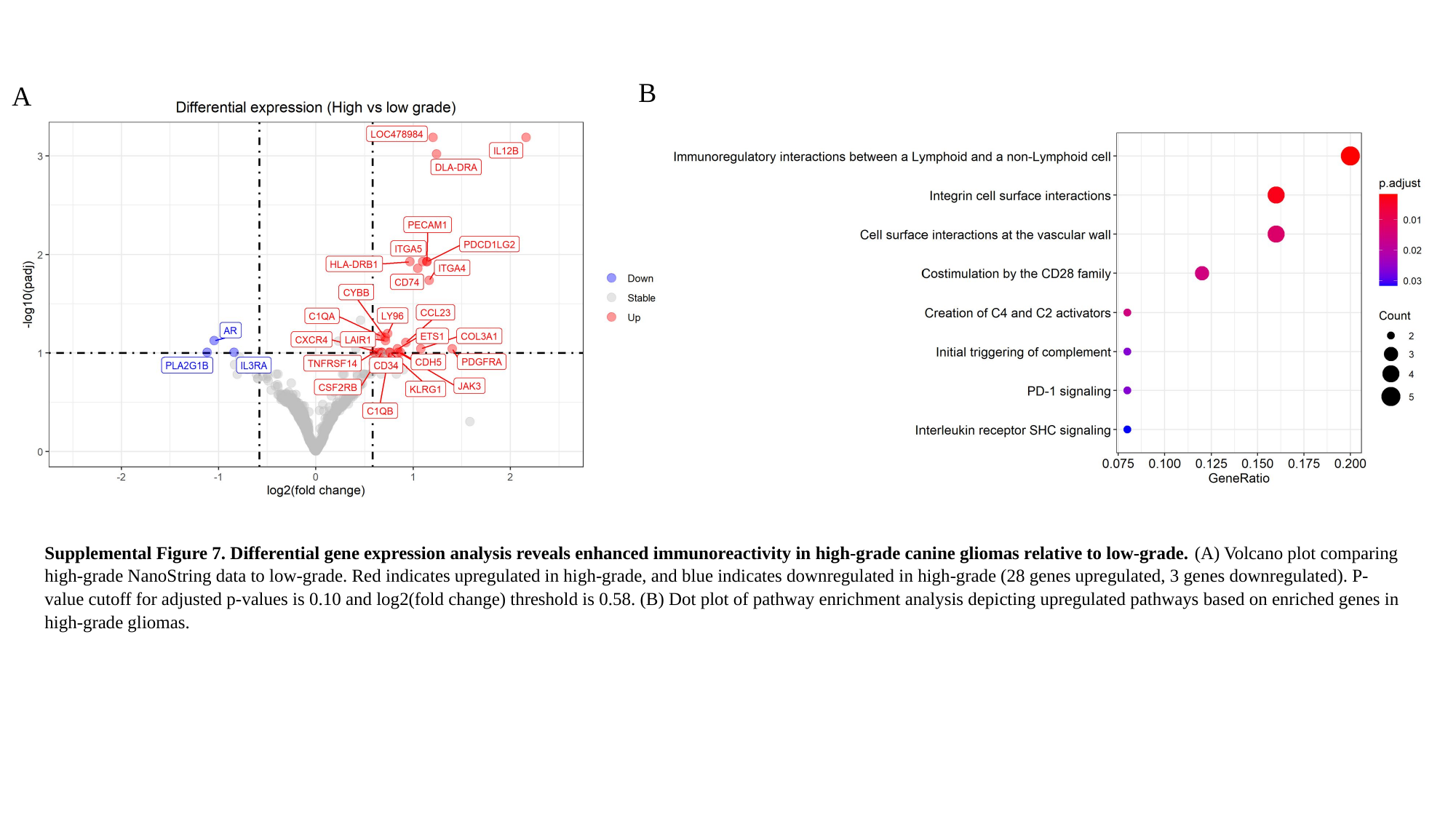

B
A
Supplemental Figure 7. Differential gene expression analysis reveals enhanced immunoreactivity in high-grade canine gliomas relative to low-grade. (A) Volcano plot comparing high-grade NanoString data to low-grade. Red indicates upregulated in high-grade, and blue indicates downregulated in high-grade (28 genes upregulated, 3 genes downregulated). P-value cutoff for adjusted p-values is 0.10 and log2(fold change) threshold is 0.58. (B) Dot plot of pathway enrichment analysis depicting upregulated pathways based on enriched genes in high-grade gliomas.

## Slide 11
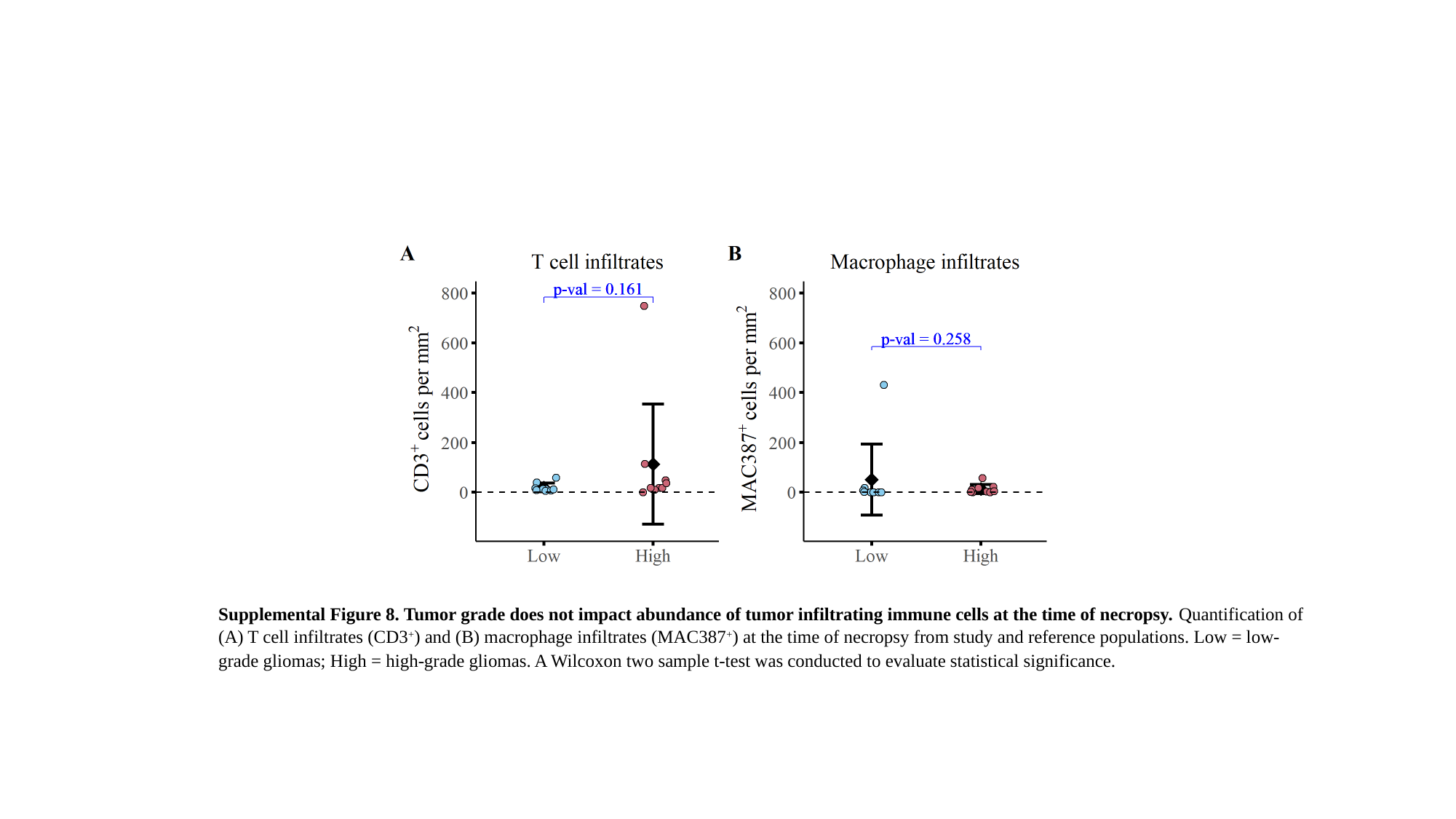

Supplemental Figure 8. Tumor grade does not impact abundance of tumor infiltrating immune cells at the time of necropsy. Quantification of (A) T cell infiltrates (CD3+) and (B) macrophage infiltrates (MAC387+) at the time of necropsy from study and reference populations. Low = low-grade gliomas; High = high-grade gliomas. A Wilcoxon two sample t-test was conducted to evaluate statistical significance.
